# Site-specific activation of the proton pump inhibitor rabeprazole by tetrathiolate zinc centres

**DOI:** 10.1038/s41557-025-01745-8

**Published:** 2025-02-20

**Authors:** Teresa Marker, Raphael R. Steimbach, Cecilia Perez-Borrajero, Marcin Luzarowski, Eric Hartmann, Sibylle Schleich, Daniel Pastor-Flores, Elisa Espinet, Andreas Trumpp, Aurelio A. Teleman, Frauke Gräter, Bernd Simon, Aubry K. Miller, Tobias P. Dick

**Affiliations:** 1 Division of Redox Regulation, German Cancer Research Center (DKFZ) and DKFZ-ZMBH Alliance, Heidelberg, Germany; 2 Faculty of Biosciences, Heidelberg University, Heidelberg, Germany; 3 Cancer Drug Development Group, German Cancer Research Center (DKFZ), Heidelberg, Germany; 4 Structural and Computational Biology Unit, European Molecular Biology Laboratory (EMBL), Heidelberg, Germany; 5 Core Facility for Mass Spectrometry and Proteomics, Zentrum für Molekulare Biologie der Universität Heidelberg (ZMBH), Heidelberg, Germany; 6 Heidelberg Institute for Theoretical Studies (HITS), Heidelberg, Germany; 7 Division of Signal Transduction in Cancer and Metabolism, DKFZ, Heidelberg, Germany; 8 Heidelberg Institute for Stem Cell Technology and Experimental Medicine (HI-STEM gGmbH), Heidelberg, Germany; 9 Division of Stem Cells and Cancer, DKFZ and DKFZ-ZMBH Alliance, Heidelberg, Germany; 10 German Cancer Consortium (DKTK), Heidelberg, Germany; 11 Faculty of Medicine, Heidelberg University, Heidelberg, Germany; 12 Max Planck Institute (MPI) for Polymer Research, Mainz, Germany; 13 Department of Molecular Biology and Biophysics, University of Connecticut Health Center, Farmington, CT USA; 14 Present Address: Drug Design Small Molecules Unit, Institute de Recherche Servier, Gif-sur-Yvette, France; 15 Present Address: KBI Biopharma SA, Plan-les-Ouates, Switzerland; 16 Present Address: Department of Pathology and Experimental Therapy, School of Medicine, University of Barcelona and Molecular Mechanisms and Experimental Therapy in Oncology Program (Oncobell), Institut d’Investigació Biomèdica de Bellvitge (IDIBELL), Barcelona, Spain

**Keywords:** Target identification, Mechanism of action, Target identification

## Abstract

Proton pump inhibitors have become top-selling drugs worldwide. Serendipitously discovered as prodrugs that are activated by protonation in acidic environments, proton pump inhibitors inhibit stomach acid secretion by covalently modifying the gastric proton pump. Despite their widespread use, alternative activation mechanisms and potential target proteins in non-acidic environments remain poorly understood. Employing a chemoproteomic approach, we found that the proton pump inhibitor rabeprazole selectively forms covalent conjugates with zinc-binding proteins. Focusing on DENR, a protein with a C4 zinc cluster (that is, zinc coordinated by four cysteines), we show that rabeprazole is activated by the zinc ion and subsequently conjugated to zinc-coordinating cysteines. Our results suggest that drug binding, activation and conjugation take place rapidly within the zinc coordination sphere. Finally, we provide evidence that other proton pump inhibitors can be activated in the same way. We conclude that zinc acts as a Lewis acid, obviating the need for low pH, to promote the activation and conjugation of proton pump inhibitors in non-acidic environments.

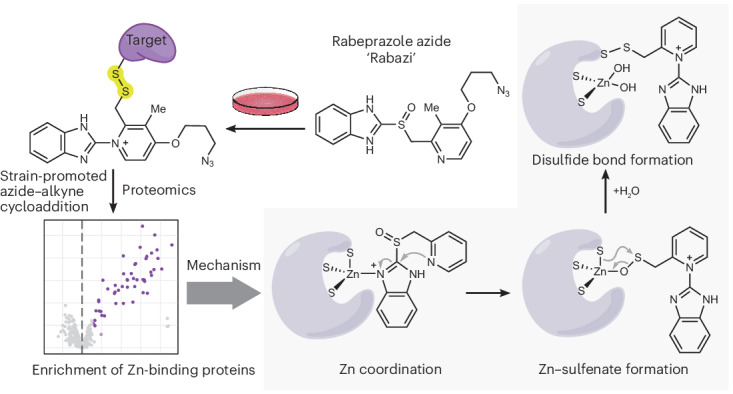

## Abstract (web summary)

Proton pump inhibitors (PPIs) are prodrugs that are activated by protonation in the highly acidic environment of the stomach lining. Now, coordination of PPIs to protein-bound zinc ions is revealed as another pathway to PPI activation. Acting as a Lewis acid, the zinc ion facilitates conjugation of the drug to zinc-coordinating cysteine residues.

## Main

**Fig. 1:**
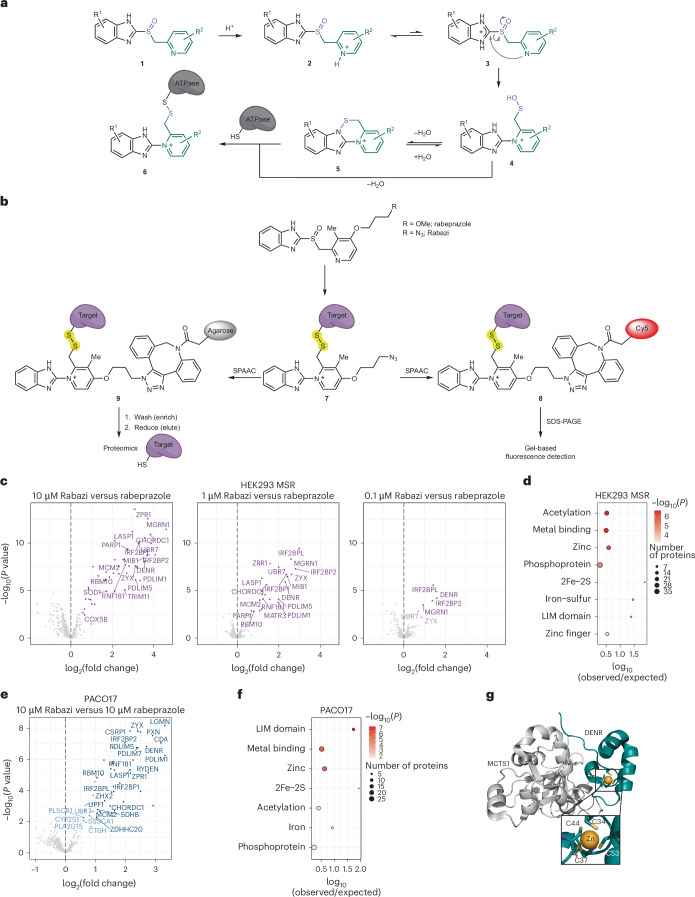
Identification of rabeprazole target proteins. **a**, Backbone structure of PPIs (**1**) and their conventional mechanism of protonation-mediated activation (**1**–**5**) and target protein conjugation (**6**). The 2-substituted pyridyl moiety is shown in green, the benzimidazolyl moiety is black and the sulfinyl group is purple. **b**, The chemoproteomic approach used to isolate (left branch) or fluorescently label (right branch) rabeprazole-reactive proteins. SPAAC, strain-promoted azide–alkyne cycloaddition; SDS-PAGE, SDS polyacrylamide gel electrophoresis. **c**, Identification of Rabazi-conjugated proteins isolated from HEK293 MSR cells treated with three different concentrations of Rabazi (left, 10 μM; middle, 1 μM; right, 0.1 μM). An empirical Bayes moderated *t*-test from the limma package was used to test for differential abundance. Significant hits are coloured purple. Proteins with at least one zinc-binding site are labelled by name. **d**, Enrichment of STRING database categories for target proteins identified from HEK293 MSR cells, relative to the total proteome. Based on a hypergeometric test using the Benjamini–Hochberg method to correct *P* values for multiple testing. **e**, Identification of Rabazi-conjugated proteins isolated from PACO17 cells treated with 10 μM Rabazi. An empirical Bayes moderated *t*-test from the limma package was used to test for differential abundance. Significant hits are coloured dark blue. Proteins with at least one zinc-binding site are labelled by name. **f**, Enrichment of STRING database categories for target proteins identified from PACO17 cells, relative to the total proteome. Based on a hypergeometric test using the Benjamini–Hochberg method to correct *P* values for multiple testing. **g**, Structure of DENR–MCTS1 complex (Protein Data Bank (PDB) no. 6MS4) with the DENR C4 zinc-binding site expanded. Source data

Proton pump inhibitors (PPIs) are listed in the World Health Organization’s List of Essential Medicines and are among the most widely used medications globally^1^. By inhibiting stomach acid production, they are used to treat peptic ulcers and gastroesophageal reflux. Six PPI variants have been approved by the US Food and Drug Administration (FDA): omeprazole, esomeprazole, lansoprazole, dexlansoprazole, pantoprazole and rabeprazole^2^. All PPIs (Fig. 1a) are based on the same chemical backbone (**1**), consisting of a 2-substituted pyridyl (green) linked to a benzimidazole moiety (black) through a sulfinyl group (purple)^3^.

By directly targeting the gastric proton pump, PPIs are the most potent inhibitors of stomach acid secretion available. Selectivity for this target is achieved because PPIs are prodrugs that are enriched and activated in highly acidic environments^4^. PPIs are administered as coated capsules that pass through the stomach intact. In the proximal small bowel, they are absorbed into the circulatory system. Reaching the epithelial lining of the stomach, PPIs have to cross two parietal cell membranes to finally enter the parietal cell canaliculi, the only space in the body with a pH below 4.0. The most basic functional group of the PPI, the pyridyl group, is rapidly protonated (p*K*_a_ ≈ 4, where *K*_a_ is the acid dissociation constant; **2**), making the molecule less membrane permeable, and thus trapping and enriching the prodrug in proximity to its target, the hydrogen potassium ATPase (H^+^/K^+^-ATPase). Proton transfer to the benzimidazolyl nitrogen (p*K*_a_ < 1; **3**) triggers an intramolecular chemical rearrangement, leading to the formation of an electrophilic sulfenic acid (**4**), which is in a condensation/hydrolysis equilibrium with the corresponding sulfenamide (**5**). In the canaliculus, surface-accessible cysteine residues of the H^+^/K^+^-ATPase react with activated PPIs (**4** or **5**) to form disulfide-linked adducts (**6**), resulting in proton pump inhibition^3,5^.

Upon entering circulation, PPIs potentially reach tissues all over the body. This raises the question of whether PPIs can be activated by alternative mechanisms, independent of very low pH. If this is the case, PPIs may have additional protein targets and effects in additional tissues/locations throughout the body. Various studies have indicated that PPIs can have effects beyond the inhibition of stomach acid production. Specifically, long-term use of PPIs may increase the risk of cardiovascular complications and kidney disorders^6–8^. These observations suggest the possibility that PPIs can be activated by other mechanisms and in other locations.

In this study, we asked if PPIs target intracellular proteins in non-acidic compartments. To this end, we used a chemoproteomics approach. We chose rabeprazole as a representative of the PPI class and equipped it with an azide handle. We incubated cultured human cells with the compound and identified cytosolic and nuclear proteins forming covalent disulfide bonds with the drug. We found that rabeprazole preferentially conjugates to zinc-binding proteins. The most highly enriched proteins all harbour a C4 zinc-binding motif in which zinc is coordinated by four cysteines. We selected one of the main target proteins, density-regulated reinitiation and release factor (DENR), for further study. We confirmed that its conjugation to rabeprazole depends on coordinated zinc, both inside cells and in vitro. We identified two of the zinc-coordinating cysteines as preferred sites of rabeprazole conjugation. We conclude that zinc promotes the activation of rabeprazole by acting as a Lewis acid, thus obviating the need for low pH. Finally, we provide evidence that other PPIs can be activated in the same way.

## Results

### Identification of rabeprazole target proteins

**Extended Data Fig. 1:**
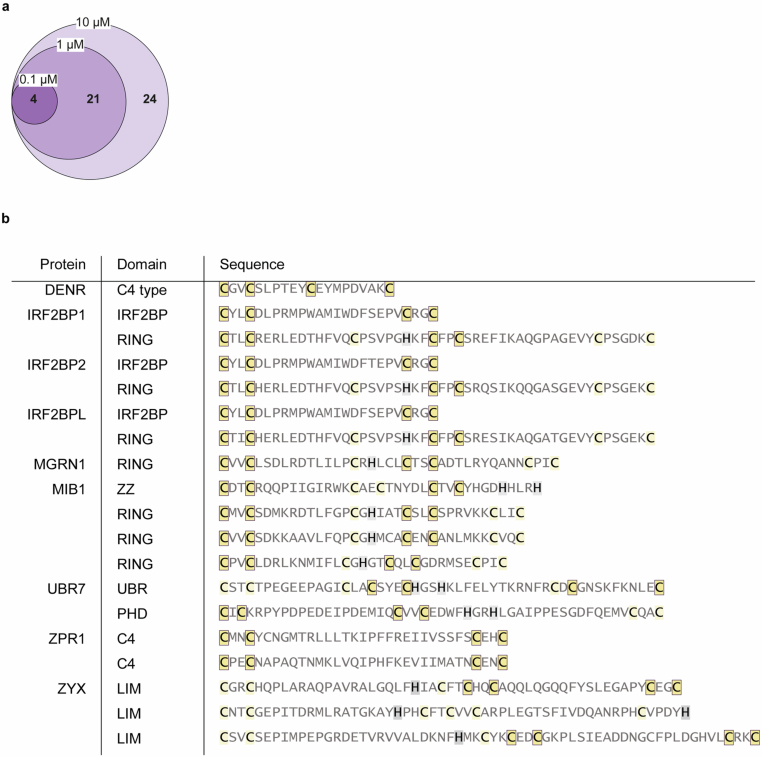
Identification of rabeprazole target proteins. (**a**) The target proteins (n = 4, n = 21 and n = 24) identified with three different concentrations of Rabazi (0.1, 1 and 10 μM) completely overlap as hierarchical nested sets. (**b**) Zinc-binding motifs of the nine most significantly enriched target proteins following treatment of cells with 1 μM Rabazi (logFC > 1.5 and FDR < 0.001). Cysteines belonging to a C4 zinc cluster are both highlighted and boxed. Cysteines and histidines belonging to other types of zinc clusters are highlighted without boxing.

To identify proteins forming covalent adducts with rabeprazole, we synthesized a rabeprazole analogue containing an azide group in place of the methoxy group (rabeprazole azide, ‘Rabazi’; Fig. 1b and Supplementary Figs. 1a,b). We incubated HEK293 MSR cells with Rabazi for 90 min, after which the cells were lysed. To avoid the use of reducing agents, which could destroy Rabazi–protein conjugates (**7**), we employed copper-free click chemistry, namely strain-promoted azide–alkyne cycloaddition^9^, to couple protein–Rabazi conjugates to either cyanine5 (Cy5; **8**) or agarose beads (**9**; Fig. 1b). In-gel visualization of Cy5-labelled proteins revealed conjugation of Rabazi to multiple proteins in HEK293 MSR cells (Supplementary Fig. 1c). As expected, the conjugates were sensitive to disulfide reduction (Supplementary Fig. 1d). Rabeprazole competed with the conjugation of Rabazi to proteins, thus confirming that Rabazi targets the same proteins as rabeprazole (Supplementary Fig. 1e). Incubating cells with Rabazi using media of different pH values did not have a notable influence on the pattern of conjugated proteins (Supplementary Fig. 1f), demonstrating that Rabazi activation occurs inside cells, independent of extracellular pH. Rabazi-conjugated proteins coupled to agarose beads were eluted by reduction and subsequently identified by mass spectrometry (MS). Incubation of cells with 10, 1 and 0.1 µM Rabazi led to the identification of 49, 25 and 4 target proteins, respectively (Fig. 1c and Supplementary Fig. 1g). Notably, these three sets of target proteins completely overlap as hierarchical nested sets (Extended Data Fig. 1a). The identified proteins are diverse in terms of subcellular location and function (Supplementary Table 1). Strikingly, however, zinc-binding proteins were found to be highly enriched (Fig. 1d). The nine most prominently enriched proteins all contained at least one C4 zinc-binding motif, in which a zinc ion is coordinated by four cysteine ligands (Extended Data Fig. 1b). This finding suggested to us the possibility that C4-coordinated zinc facilitates Rabazi activation and/or conjugation. To confirm the generality of this finding, we repeated the identification of Rabazi target proteins with a different cell line. We chose a primary human pancreatic ductal adenocarcinoma cell line (PACO17) because long-term use of PPIs has been associated with an increased risk of pancreatic cancer^10–12^. Following incubation of PACO17 cells with 10 µM Rabazi, we identified 37 target proteins (Fig. 1e and Supplementary Table 1). Of those, 22 (59%) were also identified in HEK293 MSR cells. Overall, we obtained the same high enrichment of zinc-binding proteins (Fig. 1f). To further investigate the connection between C4 zinc fingers and rabeprazole activation/conjugation, we selected density-regulated reinitiation and release factor (DENR; Fig. 1g), which was prominently trapped by rabeprazole in both cell lines, as a representative target protein for further study.

### Intracellular rabeprazole conjugation depends on Zn^2+^ binding

**Fig. 2:**
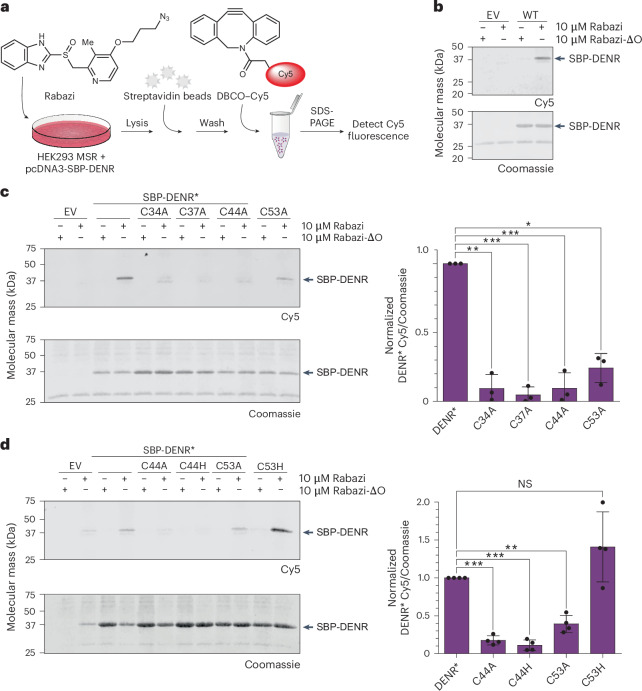
Intracellular rabeprazole conjugation depends on Zn^2+^ binding. **a**, Workflow for analysing the intracellular conjugation of Rabazi to SBP-tagged DENR expressed in HEK293 MSR cells. **b**, Rabazi conjugates to SBP-DENR in intact cells. HEK293 MSR cells transfected with SBP-DENR wild type (WT) or empty vector (EV) were treated for 15 min with 10 µM Rabazi or Rabazi thioether (Rabazi-ΔO). The lower band (25 kDa) on the Coomassie gel represents (crosslinked) streptavidin dimers released from beads. Representative blot for *n* > 7 independent experiments. **c**, Replacement of zinc-coordinating cysteines by alanines in SBP-DENR* diminishes Rabazi conjugation (left panel). Quantitation of the Cy5 signal (right panel). HEK293 MSR cells ectopically expressing SBP-DENR* or mutants were treated for 15 min with 10 µM Rabazi or Rabazi thioether (Rabazi-ΔO). Data are presented as mean ± s.d., based on *n* = 3 independent experiments. *P < 0.05, **P < 0.01, ***P < 0.001; *P* = 0.0022, 0.0001, 0.0002 and 0.012, based on a two-tailed paired *t*-test. **d**, Replacement of C44, but not of C53, by histidine in SBP-DENR* leads to the loss of Rabazi conjugation (left panel). Quantitation of the Cy5 signal (right panel). HEK293 MSR cells ectopically expressing SBP-DENR* or mutants were treated for 15 min with 10 µM Rabazi or Rabazi thioether (Rabazi-ΔO). Data are represented as mean ± s.d., based on *n* = 4 independent experiments. NS, not significant; **P < 0.01, ***P < 0.001; *P* = 0.0001, 0.0001, 0.0017 and 0.1753, based on a two-tailed paired *t*-test. Source data

To evaluate the role of the DENR C4 zinc-binding site in facilitating Rabazi conjugation, we ectopically expressed streptavidin binding peptide (SBP)-tagged DENR in HEK293 MSR cells. Treatment of these cells with Rabazi led to the formation of an SBP-DENR–Rabazi disulfide conjugate, as confirmed by affinity purification and Cy5 labelling, mediated by strain-promoted azide–alkyne cycloaddition (Fig. 2a,b). The conjugation was confirmed to be mechanism-based, because a variant of Rabazi in which the sulfoxide (–S(O)–) is replaced by a thioether (–S–) bridge (named Rabazi-ΔO) did not form any conjugates. Apart from the four cysteines involved in zinc-coordination (C34, C37, C44 and C53), DENR contains three additional cysteines (C13, C132 and C154). We therefore created the triple mutant SBP-DENR(C13A/C132A/C154A), henceforth named SBP-DENR*, which contains only zinc-coordinating cysteines. SBP-DENR* also formed a Rabazi conjugate (Fig. 2c, lane 4), showing that Rabazi conjugates to one (or more) of the zinc-coordinating cysteines. In addition, replacing any of the four zinc-coordinating cysteines by alanine strongly diminished Rabazi conjugation (Fig. 2c, lanes 5–12). This suggested that the ability of DENR to coordinate Zn^2+^ is a prerequisite for Rabazi to conjugate to one of the zinc-coordinating cysteines. Notably, the replacement of C53 by alanine was somewhat less efficient in preventing Rabazi conjugation (Fig. 2c, lane 12), potentially indicating that Zn^2+^ binding can be partially maintained in the absence of C53. In one previously reported DENR crystal structure, C53 was replaced by a histidine from a neighbouring subunit^13^. We therefore asked how replacement of C53 by histidine would affect Rabazi–DENR conjugation. In contrast to DENR*(C53A), DENR*(C53H) conjugated to Rabazi equally as well as DENR* (Fig. 2d), suggesting that H53 supports zinc binding by acting as a zinc ligand in place of C53. Thus, Rabazi–DENR conjugation in cellulo requires stably bound zinc, but not necessarily a cysteine in position 53. By comparison, we replaced C44 with histidine. DENR*(C44H) behaved like DENR*(C44A) in that it abolished Rabazi conjugation (Fig. 2d), suggesting that H44 may not be sterically compatible with Zn^2+^ coordination, or that C44 is a site of Rabazi conjugation.

### Conjugation of Rabazi to recombinant DENR depends on zinc binding

**Fig. 3:**
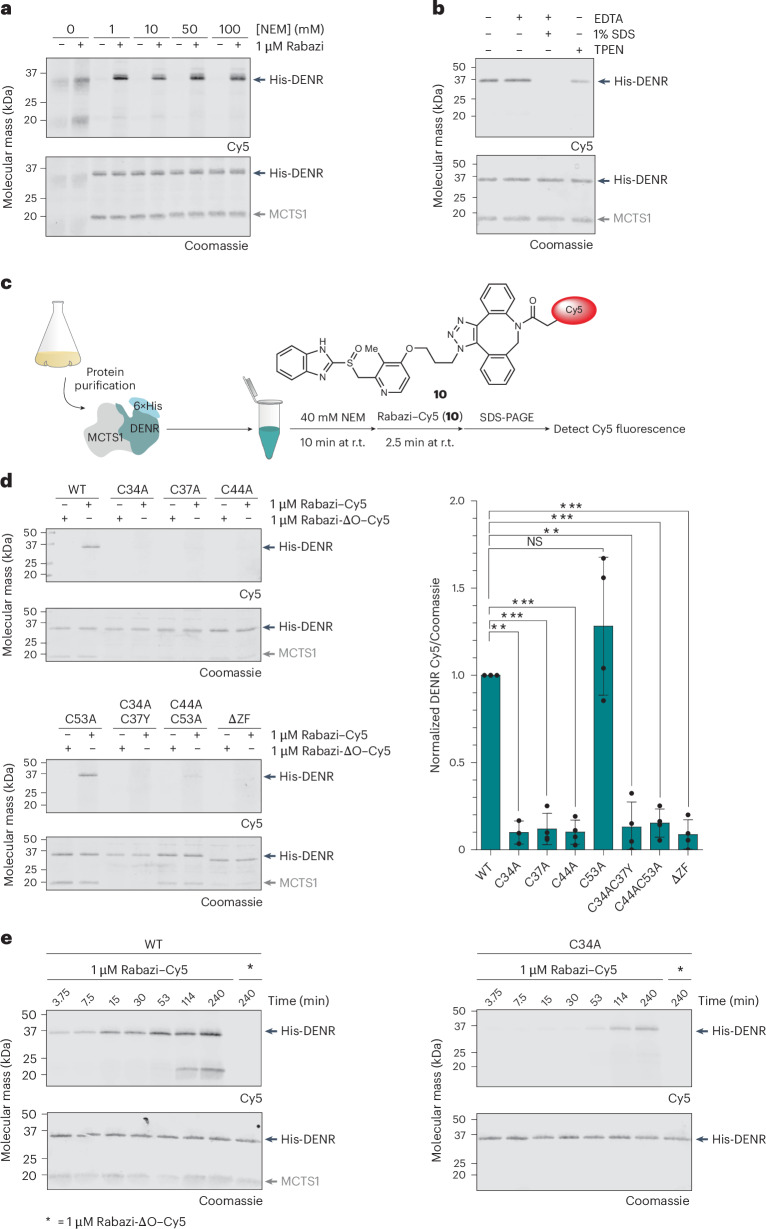
Conjugation of Rabazi to recombinant DENR depends on Zn^2+^ binding. **a**, The thiol alkylating agent NEM improves the detection of Rabazi-conjugated recombinant WT DENR in the Cy5-based gel assay. NEM does not interfere with Rabazi conjugation even when applied at very high concentration (100 mM). Representative blot of *n* = 2 independent experiments. **b**, Conjugation of Rabazi to recombinant DENR is insensitive to EDTA (2.5 mM), diminished by the stronger metal chelator TPEN (2.5 mM) and completely abolished by the combination of EDTA (2.5 mM) and SDS (1%). The experiment was conducted in the presence of NEM. Representative blot of *n* = 2 independent experiments. **c**, Workflow for assessing the interaction between Rabazi and the recombinant His-DENR–MCTS1 complex. **d**, Mutation of the zinc-coordinating cysteines in DENR diminishes Rabazi conjugation, with the exception of C53A (left panel). Quantitation of the Cy5 signal (right panel). Data are represented as mean ± s.d., based on *n* = 4 (C34A, *n* = 3) independent experiments. **P < 0.01, ***P < 0.001; NS, not significant; *P* = 0.0018, 0.0003, 0.0001, 0.2494, 0.0012, 0.0002 and 0.0002, based on a two-tailed paired *t*-test. **e**, Recombinant WT DENR conjugates to Rabazi more rapidly than DENR(C34A). Representative blot of *n* = 2 independent experiments. Source data

**Extended Data Fig. 2:**
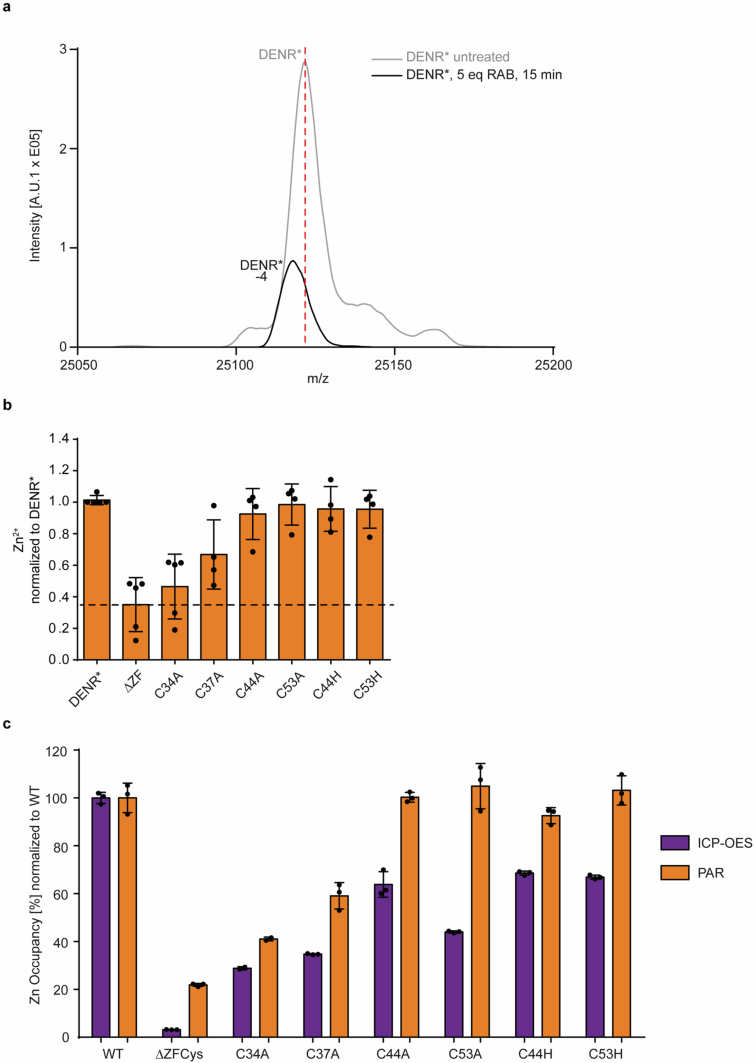
Conjugation of Rabazi to recombinant DENR depends on zinc binding. (**a**) Intact protein MS of recombinant DENR* following incubation with a 5-fold molar excess of rabeprazole for 15 min at r.t. The observed mass shift (–4 Da) indicates the formation of two intramolecular disulfide bonds in the presence, but not in the absence of rabeprazole. (**b**) Quantification of Zn^2+^ bound to recombinant His-DENR:MCTS1 and respective cysteine mutants. Zn^2+^ was released from 10 µM recombinant protein by incubation with 4 M guanidinium chloride (GdnHCl) and 100 mM NEM. PAR was added to a final concentration of 100 µM and formation of the zinc-PAR complex was quantified spectrophotometrically. Data are represented as mean ± s.d., based on n = 4 (C37A, C44A, C53A, C44H, C53H) or n = 5 (DENR*, C34A, ΔZF) independent experiments with n = 3 technical replicates each. (**c**) Zinc occupancy of His-DENR:MCTS1 and respective mutants as determined by PAR and ICP-OES. Data are represented as mean ± s.d., based on *n* = 3 (C34A: *n* = 2) technical replicates each. Source data

For further analysis, we recombinantly expressed His-tagged human WT DENR in *Escherichia coli*. To stabilize His-DENR, we co-expressed it with its natural binding partner MCTS1 and purified the recombinant His-DENR–MCTS1 complex. We found that Rabazi conjugation to recombinant DENR leads to a rather weak and diffuse band in the gel-based Cy5 assay unless a thiol-reactive alkylating agent like *N*-ethylmaleimide (NEM) was added together with Rabazi. The presence of NEM at concentrations of 1 mM or higher intensified and sharpened the band representing the DENR–rabeprazole conjugate, and also largely prevented labelling of MCTS1 (Fig. 3a). This effect can be expected, because alkylation of free thiols prevents secondary reactions. Without the alkylating agent, any Rabazi–DENR disulfide conjugate that is initially formed may engage in thiol–disulfide exchange reactions with other thiol groups, either those outside the zinc-binding site, those becoming available upon dissociation of the Zn^2+^ ion (vide infra) or those available on the surface of MCTS1. These secondary reactions cause either the release of Rabazi from the protein or its transfer to secondary sites, thus rationalizing the observation of weak, diffuse and non-specific bands (Fig. 3a, lanes 1 and 2). Secondary displacement of conjugated rabeprazole by neighbouring protein thiols should lead to the formation of intramolecular disulfide bonds. Indeed, we found that DENR* forms intramolecular disulfide bonds in the presence of rabeprazole, but not in its absence (Extended Data Fig. 2a).

On the basis of these findings, we proceeded with the combination of NEM and Rabazi. First, we investigated the effect of removing zinc from recombinant WT DENR. While the metal chelator ethylenediaminetetraacetic acid (EDTA) was inefficient, the stronger chelator *N*,*N*,*N*′,*N*′-tetrakis(2-pyridinylmethyl)-1,2-ethanediamine (TPEN) partially suppressed Rabazi conjugation (Fig. 3b). Importantly, the combination of EDTA and sodium dodecyl sulfate (SDS) completely abolished Rabazi conjugation (Fig. 3b), supporting the notion that a structurally intact zinc-binding site is crucial for the reaction to take place.

Next, we exposed the recombinant protein to NEM and then added a ‘pre-clicked’ Rabazi–Cy5 conjugate (**10**; Fig. 3c and Supplementary Fig. 2a), generating a robust fluorescent band. Mutation of either C34, C37 or C44 abolished conjugation almost completely (Fig. 3d, upper panel). However, unlike in cellulo (Fig. 2c), the mutation of C53 did not interfere with conjugation (Fig. 3d, lower panel). One explanation is that recombinant MCTS1-stabilized DENR is able to maintain Zn^2+^ binding in the absence of C53 (ref. ^13^). Consistent with the role of the other zinc-coordinating cysteines, double mutations (C34A/C37Y or C44A/C53A) and the quadruple mutation (C34A/C37Y/C44A/C53A, named ΔZF) abolished Rabazi conjugation (Fig. 3d, lower panel). A kinetic comparison confirmed that zinc-proficient WT DENR is much faster in conjugating Rabazi–Cy5 than the zinc-deficient mutant DENR(C34A) (Fig. 3e). A quantitative zinc assay showed that single mutants C34A and C37A are largely zinc-deficient, while single mutants C44A and C53A partially or largely retain zinc (Extended Data Fig. 2b,c), presumably because the core CXXC motif (C34 and C37) of the cluster is still intact.

### The C4 zinc cluster tends to disengage one of its ligands

**Extended Data Fig. 3:**
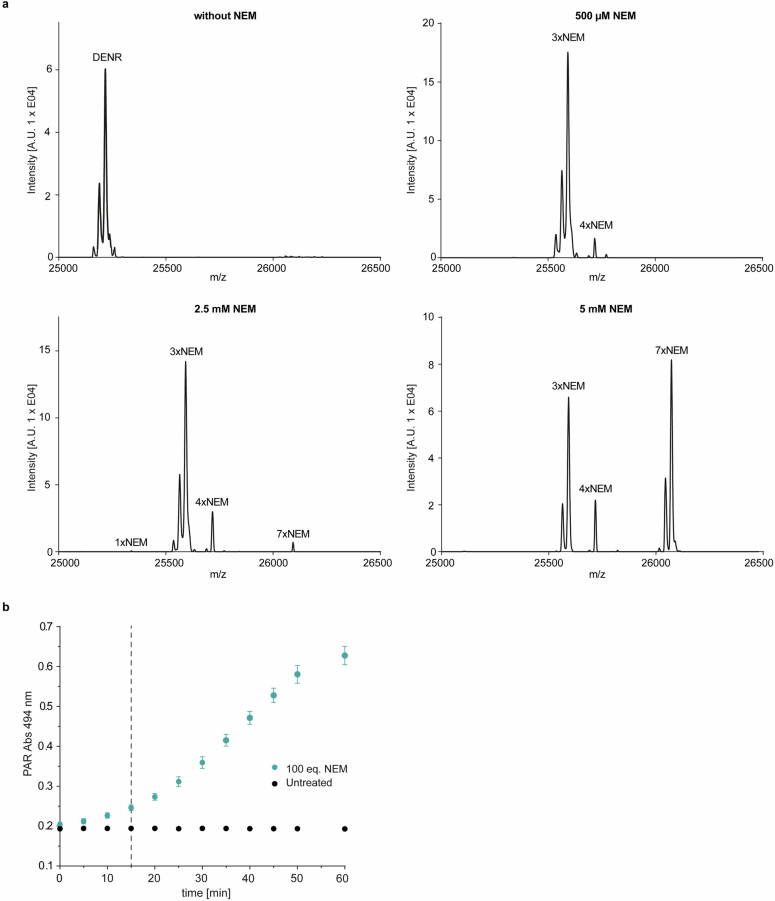
The C4 zinc cluster tends to disengage one of its ligands. (**a**) Intact protein MS of recombinant DENR* (5 μM) following incubation with different concentrations of NEM (0, 0.5, 2.5 and 5 mM) for 15 min at r.t. (**b**) Release of Zn^2+^ ions from His-DENR* (7.5 µM), induced by a 100-fold molar excess of NEM, as monitored by chelation with 4-(2-pyridylazo)resorcinol (PAR; 100 µM). The 15 min time point relevant for comparison with (a) is highlighted by a dashed vertical line. Based on the mean ± s.d. of *n* = 3 technical replicates. Source data

To better understand the dynamics of the DENR zinc-binding site in the presence of thiol modifying agents, we performed intact protein MS. After incubating WT DENR for 15 min with a 100-fold molar excess of NEM, most of the protein was found conjugated to three NEM molecules (Extended Data Fig. 3a, upper panels), matching the number of free (non-zinc-coordinating) cysteines. This suggested that the four zinc-coordinating cysteines are largely protected against NEM adduction. However, a smaller fraction of DENR is found conjugated to four NEM molecules, indicating that one of the four zinc-coordinating cysteines dissociated and subsequently became alkylated. Further increasing the concentration of NEM (500-fold and 1,000-fold molar excess) caused the relative abundance of the ×4 NEM-modified peptide to increase and a ×7 NEM (fully alkylated) peptide to appear (Extended Data Fig. 3a, lower panels). Notably, ×5 NEM-modified or ×6 NEM-modified peptides could not be identified in any of these experiments. These findings suggest that the zinc cluster is prone to dissociate one of its cysteine ligands, but then remains relatively stable as a trithiolate zinc cluster. However, any further alkylation leads to the disintegration of the whole cluster, thus causing full alkylation (×7 NEM) and Zn^2+^ release. Indeed, DENR increasingly released Zn^2+^ into the supernatant under the given conditions (Extended Data Fig. 3b).

Together, these observations suggested a model of how rabeprazole gains access to the protein-bound Zn^2+^ ion: if one of the cysteine ligands of the tetrathiolate cluster dissociates (presumably C53) and leaves behind a relatively stable trithiolate cluster, then rabeprazole can occupy the liberated coordination site by engaging its benzimidazole nitrogen. Moreover, the fact that NEM, even at high molar excess, does not compete with rabeprazole conjugation (Fig. 3a) strongly suggests that one (or more) of the three most stably coordinated cysteines (C34, C37, C44) must be the conjugation site for rabeprazole.

### Rabeprazole conjugates to zinc-coordinating cysteines 44 and 34

**Extended Data Fig. 4:**
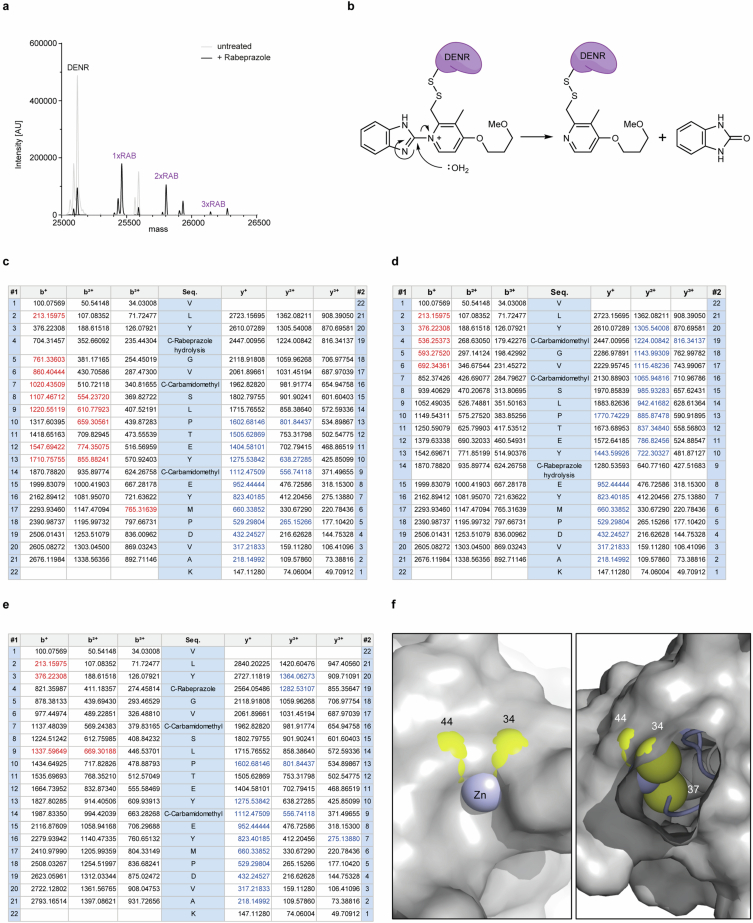
Rabeprazole conjugates to zinc-coordinating cysteines 44 and 34. (**a**) Intact protein MS of recombinant DENR*-MCTS1 complex incubated with a 5-fold molar excess of rabeprazole for 15 min. DENR predominantly conjugates one molecule of rabeprazole. (**b**) Hydrolysis of protein rabeprazole conjugates: the electrophilic central carbon atom of the benzimidazolyl group is susceptible to nucleophilic attack by water leading to loss of benzimidazolone. (**c**) List of fragment matches from scan number 24412 (XCorr = 5.14) indicating the presence of hydrolyzed rabeprazole on C34. Identified y-ions are marked blue and b-ions are marked red. A consecutive series of y-ions and b-ions cover the peptide sequence from the C- and N-terminal end, respectively. (**d**) List of fragment matches from scan number 24206 (XCorr = 3.51) indicating the presence of hydrolyzed rabeprazole on C44. Identified y-ions are marked blue and b-ions are marked red. A consecutive series of y-ions and b-ions cover the peptide sequence from the C- and N-terminal end, respectively. (**e**) List of fragment matches from scan number 24585 (XCorr = 3.42) indicating presence of non-hydrolyzed rabeprazole on C34. Identified y-ions are marked blue and b-ions are marked red. A consecutive series of y-ions covers the peptide sequence from the C-terminal end. (**f**) Accessibility of the DENR zinc cluster after dissociation of C53. Left panel: Surface rendering. Only the sulfur atoms of C34 and C44 are directly accessible on the surface. Right panel: Sectional view through the volume model of DENR, with the clipping plane just above the zinc cluster. The sulfur atom of C37 can be seen to be buried inside the protein. Source data

To identify the target cysteine(s) of rabeprazole, we first confirmed its conjugation to DENR by intact protein MS. Following incubation of DENR* with rabeprazole for 15 min at room temperature, DENR* was predominantly conjugated to one rabeprazole molecule, exhibiting the expected mass shift (Extended Data Fig. 4a).

**Fig. 4:**
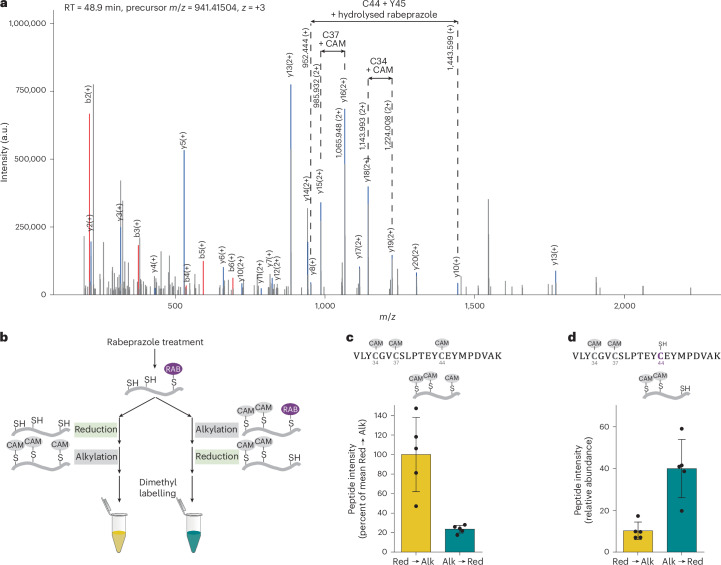
Rabeprazole conjugates to zinc-coordinating cysteines 44 and 34. **a**, Representative MS/MS spectrum of DENR peptide 31–52, demonstrating the presence of hydrolysed rabeprazole on C44. The corresponding list of fragment matches is provided in Extended Data Fig. 4d. a.u., arbitrary units; CAM, carbamidomethyl; RT, retention time; m/z, mass-to-charge ratio. **b**–**d**, Differential alkylation suggests C44 as the preferred conjugation site of rabeprazole (RAB). **b**, Workflow used to identify the preferred target cysteine of rabeprazole on DENR*. **c**, Analysing tryptic peptide 31–52, containing C34, C37 and C44, the fully alkylated form is predominantly found when reduction (Red) precedes alkylation (Alk). **d**, By contrast, the doubly alkylated form (lacking alkylation on C44) is predominantly found when alkylation (Alk) precedes reduction (Red). Data in bar graphs are represented as mean ± s.d., based on *n* = 2 independent experiments with 2 and 3 technical replicates. Source data

We then aimed to directly detect the specific cysteine–rabeprazole adduct by liquid chromatography with tandem MS (LC-MS/MS). Rabeprazole–protein conjugates can be expected to be prone to hydrolysis at the pyridinium–benzimidazolyl C–N bond, thus generating lower-molecular-weight pyridine conjugates (Extended Data Fig. 4b). Therefore, we searched for both intact and hydrolysed forms of conjugated rabeprazole. Using bottom-up proteomics, we identified the hydrolysed form of rabeprazole on cysteines C44 (Fig. 4a and Extended Data Fig. 4c) and C34 (Extended Data Fig. 4d), and the non-hydrolysed form on C34 (Extended Data Fig. 4e). Conjugation of rabeprazole to C34 and C44, but not to C37, can be explained by the relative accessibility of the sulfur atoms in the zinc cluster (Extended Data Fig. 4f).

As a complementary strategy, and to estimate the conjugation efficiency, we used a differential alkylation approach. Again, DENR* was exposed to rabeprazole (25 µM; fivefold excess) for 15 min at r.t. Immediately thereafter, the reaction was quenched by protein precipitation and the denatured protein was subjected to differential alkylation (that is, reduction followed by alkylation, or alkylation followed by reduction). In the first approach, disulfides resulting from rabeprazole binding are reduced and hence produce triply alkylated peptides, while in the second approach, they do not (Fig. 4b, compare left and right paths). Comparing intensities of the triply alkylated peptides under the two conditions indicates approximately 80% occupancy of binding by rabeprazole (Fig. 4c) and confirms VLYCGVCSLPTEYCEYMPDVAK to be the main target of rabeprazole. To identify the exact binding site(s), we compared the intensities of doubly alkylated peptides. In line with our previous observation that C44 is required for Rabazi conjugation, we identified this residue as a binding site of rabeprazole (Fig. 4d).

### Rabeprazole causes major conformational changes in DENR

To gain structural insight into the interaction between rabeprazole and DENR, we attempted to crystallize the DENR–MCTS1–rabeprazole complex with no success. In addition, soaking of rabeprazole into DENR–MCTS1 crystals led to cracking, suggesting that rabeprazole conjugation leads to major structural disruptions.

**Fig. 5:**
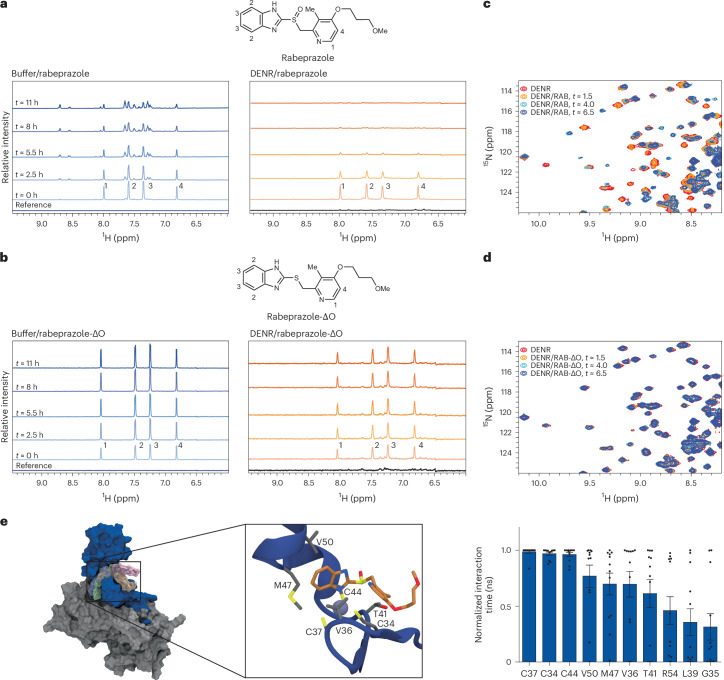
Rabeprazole causes major conformational changes in DENR. **a**, Time (*t*) series of ^1^H spectra of rabeprazole in buffer, in the absence (left) and presence (right) of the ^15^N-labelled DENR^26–98^–MCTS1 complex. **b**, Time series of ^1^H spectra of rabeprazole thioether (rabeprazole-ΔO) in buffer, in the absence (left) and presence (right) of the ^15^N-labelled DENR^26–98^–MCTS1 complex. **c**, A 2D ^1^H–^15^N HSQC spectrum of the ^15^N-labelled DENR^26–98^–MCTS1 complex in the presence of rabeprazole (RAB) collected over time. **d**, A 2D ^1^H–^15^N HSQC spectrum of the ^15^N-labelled DENR^26–98^–MCTS1 complex in the presence of rabeprazole thioether (RAB-ΔO) collected over time. **e**, Hydrophobic interactions promote the initial coordination of rabeprazole to the zinc cluster. The left shows the structure of the DENR–MCTS1–rabeprazole complex with four possible rabeprazole configurations (highlighted in transparent colours) aligned on the zinc cluster. The inset shows one rabeprazole configuration obtained from MD simulations together with interacting DENR residues. The right shows DENR residues interacting with rabeprazole based on MD simulations of four initial rabeprazole configurations. For every trajectory snapshot, residues with an atom–atom distance below 3.5 Å were classified as interacting. Only snapshots with reactive configurations (pyridine N to benzimidazole C distance within 20% of the sum of van der Waals radii) were considered. Data are presented as mean ± s.e.m., based on *n* = 3 simulations of four different configurations. Source data

**Extended Data Fig. 5:**
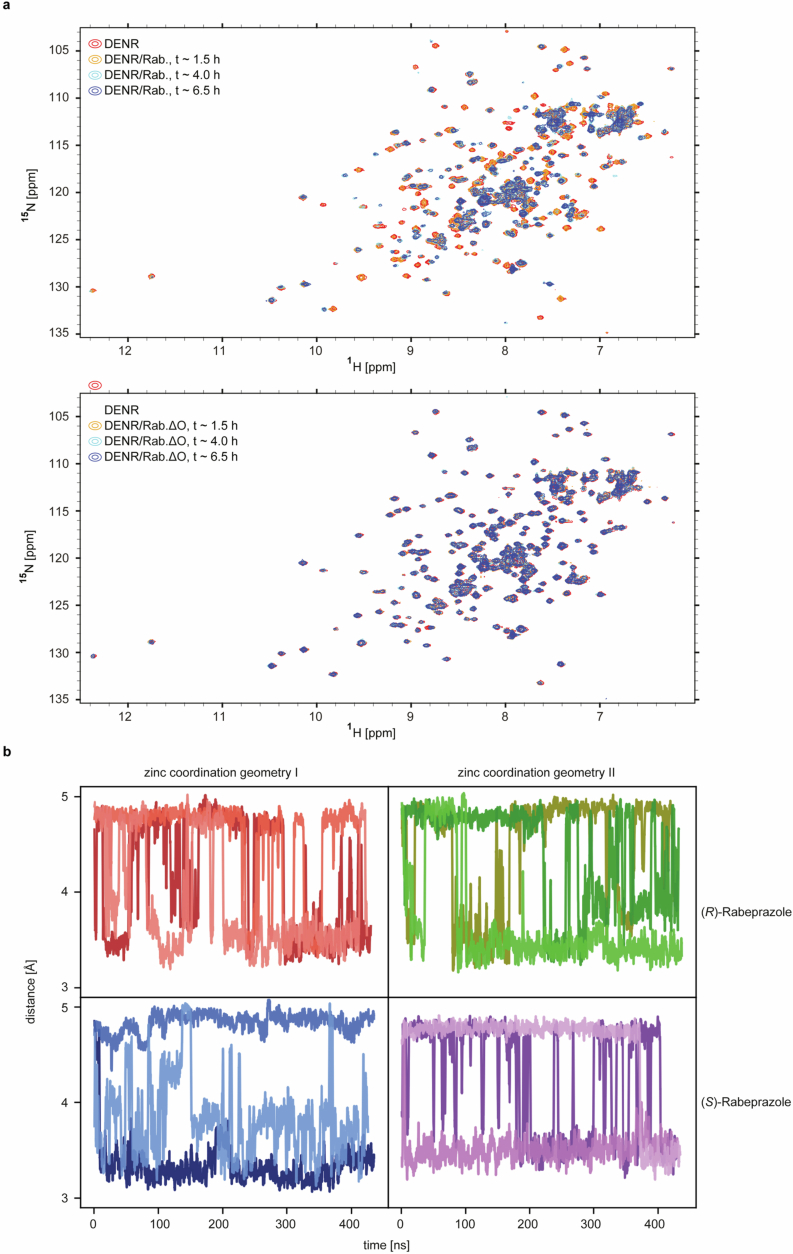
Rabeprazole causes major conformational changes in DENR. (**a**) Complete 2D ^1^H-^15^N HSQC spectrum for the experiment shown in main Figs. 5b and 5d. (**b**) Distance between the pyridine nitrogen and the benzimidazole carbon. MD simulations of the DENR-MCTS1-rabeprazole complex with either (R)- (upper panels) or (S)-rabeprazole (lower panels), with the benzimidazole nitrogen coordinated between C34 and C37 (coordination geometry I, left panels) or between C37 and C44 (coordination geometry II, right panels). Individual trajectories are shown for every initial configuration (*n* = 3 technical replicates). Source data

We then turned to NMR spectroscopy for its ability to monitor structural changes in solution. Using one-dimensional (1D) proton (^1^H) experiments, we tracked perturbations in the small molecules rabeprazole and rabeprazole-ΔO at neutral pH over time. In the absence of DENR, rabeprazole underwent slow spontaneous activation in aqueous buffer over many hours (Fig. 5a, left panel). However, in the presence of DENR, rabeprazole ^1^H signals disappeared completely over a shorter time frame (Fig. 5a, right panel). This is consistent with conjugation to DENR, due to an increase in the apparent molecular weight of the molecule and thus in a loss in signal intensity in the filtered 1D experiment. By contrast, ^1^H signals associated with rabeprazole-ΔO did not change over time, independent of the presence or absence of DENR (Fig. 5b). In parallel, we monitored the backbone amide (^1^H–^15^N) chemical shifts of DENR to determine whether rabeprazole caused conformational changes consistent with a chemical reaction. Using a series of two-dimensional (2D) ^1^H–^15^N heteronuclear single quantum coherence (HSQC) experiments collected over hours, we found that DENR exhibited time-dependent chemical shift perturbations of the amide peaks during incubation with rabeprazole, but not with rabeprazole-ΔO (Fig. 5c,d and Extended Data Fig. 5a). This was evidenced by the appearance of peaks at new positions, and the concomitant disappearance of those corresponding to unperturbed (‘free’) DENR. These observations demonstrate a change in the protein’s chemical environment (and therefore structure) as a function of time, indicative of a chemical transformation and consistent with the 1D experiments above.

Finally, we performed docking and molecular dynamics (MD) experiments. By docking rabeprazole to a DENR–MCTS1 complex with an exposed zinc-binding site, we identified preferred configurations in which the benzimidazolyl nitrogen engages the free coordination site of Zn^2+^. Depending on rabeprazole chirality and coordination geometry, the identified configurations fell into four subsets, with two possible orientations for the benzimidazole moiety. Starting from all four complexes, MD sampled stable configurations within nanoseconds (Fig. 5e, left panel). These display frequent contacts between the pyridyl nitrogen and the benzimidazolyl carbon (Extended Data Fig. 5b), as required for drug activation. The diverse ensemble of structures primed for activation shares a common set of hydrophobic interactions between DENR and rabeprazole. Apart from the cysteines of the zinc cluster, rabeprazole mainly interacts with V36, T41, M47 and V50, which together form a binding cleft that either stabilizes the benzimidazoyl in plane with the Zn–N coordinative bond or harbours the pyridyl ring, depending on the coordination geometry (Fig. 5e, middle and right panels). Together, these results support the notion that the benzimidazolyl and pyridyl rings of rabeprazole engage in hydrophobic interactions that are likely to promote the initial coordination of rabeprazole to the zinc cluster.

### All PPI variants react with the DENR C4 zinc finger

**Fig. 6:**
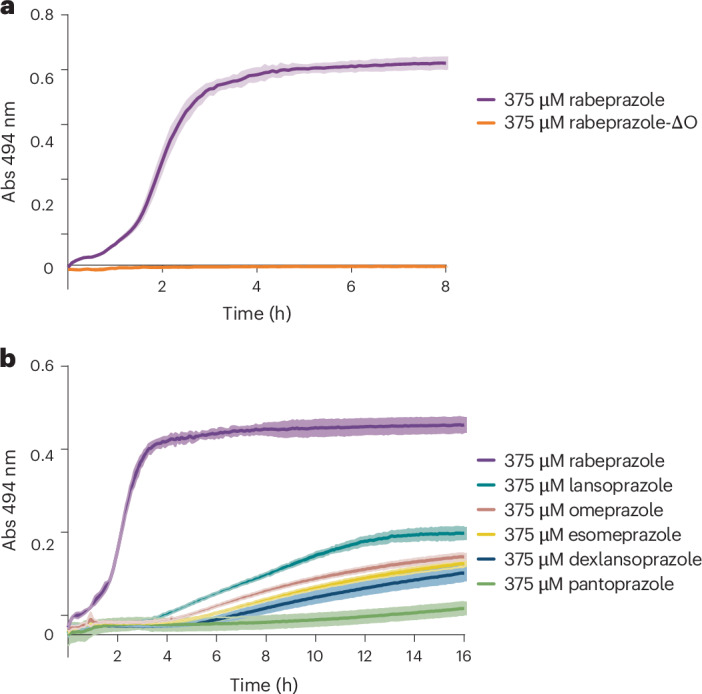
All PPI variants react with the DENR C4 zinc finger. **a**, Release of Zn^2+^ ions from His-DENR* (7.5 µM), in response to a 50-fold molar excess of rabeprazole or rabeprazole thioether (rabeprazole-ΔO), as monitored by chelation with 4-(2-pyridylazo)resorcinol (PAR; 100 µM). Based on the mean of *n* = 3 technical replicates. Shading indicates s.d. **b**, Same experiment as in **a**, testing the release of Zn^2+^ ions from DENR in response to all six FDA-approved PPIs. Based on the mean of *n* = 3 technical replicates. Shading indicates s.d. Source data

Finally, we asked if the observations made with rabeprazole also apply to other PPIs. Conjugation of a PPI to one of the zinc-coordinating cysteines is expected to destabilize the whole cluster, and therefore should lead to the release of free Zn^2+^ ions over time. Indeed, incubating DENR with rabeprazole led to the release of zinc (Fig. 6a and Supplementary Fig. 3a). By contrast, incubation with rabeprazole-ΔO did not release any zinc, even after very long incubation times, confirming that rabeprazole-induced Zn^2+^ release is a mechanism-based effect (Fig. 6a). We then asked if and to what extent the other FDA-approved PPIs would release Zn^2+^ ions from DENR. We found that all of them induce zinc release, albeit less efficiently than rabeprazole (Fig. 6b). The release of Zn^2+^ from DENR by all FDA-approved PPIs was additionally demonstrated by using Zincon, a chromophoric Zn^2+^ indicator of lower affinity, to confirm that zinc ejection is not a chelator-driven process, but rather a reflection of decreasing Zn^2+^ ion affinity (Supplementary Fig. 3b). These results support the notion that all PPIs share the ability to interact and react with C4 Zn fingers.

## Discussion

In this work, we used a chemoproteomic approach to identify previously unknown target proteins of rabeprazole, which we chose as a representative for the PPI class of drugs. We identified cytosolic C4 zinc fingers as activators and targets of rabeprazole and, most likely, of PPIs in general.

**Fig. 7:**
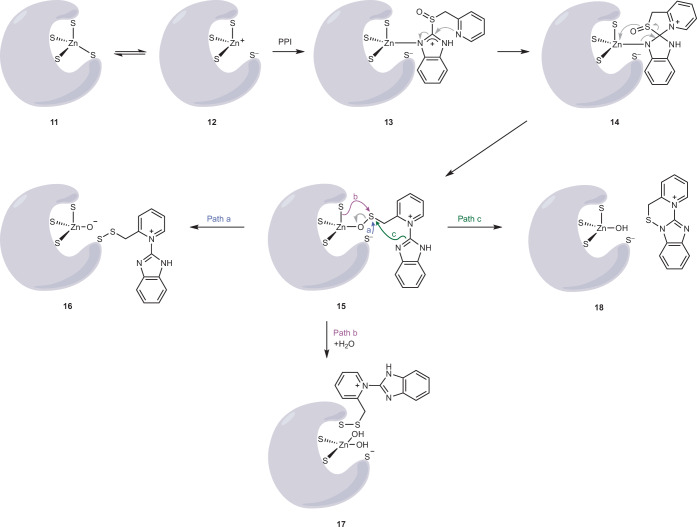
Proposed mechanism for the reaction of a PPI with a C4 zinc finger. The possible pathways promoting PPI conjugation to a C4 zinc cluster cysteine are shown. Following binding and activation (**11**–**15**), the PPI may either conjugate to the cysteine that first dissociated from the zinc (**16**; path a); conjugate to a zinc-coordinated cysteine (**17**; path b); or produce a sulfenamide intermediate, which can react with any nearby cysteine (**18**, path c). The results obtained in this work support path b.

On the basis of our findings, we propose the following mechanism for PPI activation and conjugation (Fig. 7): within a C4 zinc-binding site, one of the cysteine ligands is in rapid equilibrium between an associated (**11**) and disassociated (**12**) state, transiently opening up a coordination site on the zinc atom. A PPI should then be able to form a zinc complex (**13**) via the benzimidazolyl nitrogen, analogous to the imidazolyl in protein histidine side chains. The zinc atom functions as a Lewis acid, playing a role analogous to protonation in the classical PPI activation pathway. Indeed, the zinc-coordinated benzimidazolyl nitrogen is the same one that is protonated in the classical activation pathway, and the ensuing chemical rearrangements are similar. Nucleophilic attack of the pyridyl nitrogen leads to spirocycle **14**, with rearomatization of the benzimidazolyl producing zinc sulfenate **15**. For the final step, there are three possibilities: (1) cleavage of the sulfenate (path a, in blue) by the labile (dissociated) cysteine leading to disulfide **16**; (2) reaction of the sulfenate with one of the remaining zinc-coordinated thiols (path b, in purple) leading to disulfide **17**; and (3) formation of a reactive sulfenamide in close proximity to the zinc-binding site (**18**; path c, in green), which may then react with any nearby cysteines (not shown).

In our paper we present several observations in line with our mechanistic interpretation. First, the conjugation of rabeprazole to DENR depends on zinc coordination. Denaturation of DENR, leading to Zn^2+^ dissociation, abolishes rabeprazole conjugation. Second, conjugation of rabeprazole to zinc-coordinating cysteines is not intercepted by NEM, even when added in 10^5^-fold molar excess, suggesting that activation and conjugation take place rapidly within the zinc coordination sphere. This speaks in favour of path b, which couples cysteine ligand dissociation to disulfide bond formation in a formal 1,2-elimination reaction (**17**). Third, in line with the notion that rabeprazole targets a zinc-coordinating cysteine, rabeprazole conjugation is observed to destabilize the zinc cluster, as evidenced by Zn^2+^ release and disappearance of DENR NMR signals at later time points, suggesting increasing denaturation.

**Extended Data Fig. 6:**
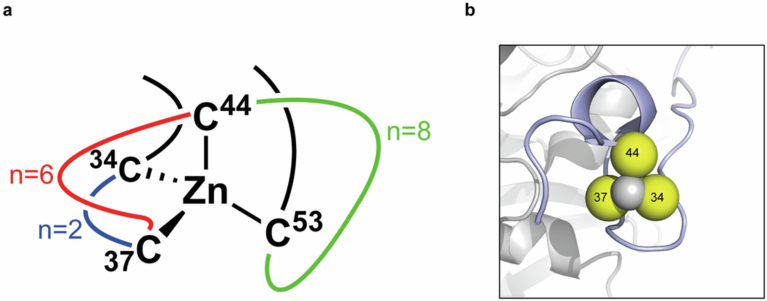
Structural organization of the DENR zinc-binding site. (**a**) Topology of the DENR zinc-binding site. Blue: C34-C37 loop; Red: C37-C44 loop; Green: C44-C53 loop. The loop lengths (n) are indicated. (**b**) Structure of the zinc- binding site of DENR (based on PDB entry 6MS4) not including the C-terminal part beyond P48, to illustrate the appearance of the cluster after dissociation of C53. The DENR binding partner MCTS1 is shown in the background (shaded in gray). Please also see the corresponding surface rendering (Extended Data Fig. 4f), showing differences in the surface exposure of the sulfur atoms.

Intact protein MS of DENR exposed to the thiol alkylating agent NEM revealed that the C4 zinc cluster is prone to open at one position, maintaining a relatively stable C3 zinc cluster. In the C4 cluster, an N-terminal CXXC core motif (C34 and C37) is followed by a cysteine that is relatively close along the protein sequence (C44), and one that is more distal (C53; Extended Data Fig. 6a). Considering that CXXC motifs have a high affinity for Zn^2+^ and that Cys pairs with greater sequence separation typically bind Zn^2+^ less tightly than pairs with closer proximity^14^, the dissociation of the most distal C-terminal cysteine (C53) appears most likely. This notion is also supported by X-ray crystallography, which shows the C53 sulfur–zinc bond to be longer than those formed between the other cysteines and the Zn^2+^ ion^15^. Furthermore, there is an exposed loop between C44 and C53, centred around P48. If the C-terminal part of DENR would move or swing out at this position, a compact structural unit in which zinc is coordinated by just C34, C37 and C44 would be exposed (Extended Data Fig. 6b). This idea is also supported by molecular docking and MD (Fig. 5e and Extended Data Fig. 5b), which suggest that hydrophobic side-chain interactions promote the positioning of the drug next to the exposed zinc cluster. Of note, the coordination of a drug’s benzimidazole moiety to a zinc cluster, analogous to histidine coordination, is not without precedent^16,17^.

Our data show that C44 and C34, but not C37, are conjugation sites of rabeprazole. This observation is most likely explained by the relative accessibility of the sulfur atoms in the C3 zinc cluster. Following the opening of the coordination site for rabeprazole (i.e., detachment of C53), only the sulfur atoms of C34 and C44 are surface exposed, while the sulfur atom of C37 is inaccessible (Extended Data Fig. 4f). Our data hint at the possibility that C44 is the preferred primary site of covalent attachment, and that rabeprazole may secondarily transfer between C44 and C34 by thiol–disulfide exchange. Assuming that the strongest contribution to zinc binding is provided by the CXXC motif, it seems plausible that the zinc-bound sulfenate preferentially reacts with C44 rather than with the more tightly bound CXXC cysteines (corresponding to path b in Fig. 7).

Our findings raised the question if the other FDA-approved PPIs (omeprazole, esomeprazole, lansoprazole, dexlansoprazole and pantoprazole) also react with C4 zinc fingers. Given their shared benzimidazole core structure, this appears to be likely. Indeed, all of them destabilized the DENR zinc finger (Fig. 6b), albeit with different kinetics (rabeprazole ≫ lansoprazole > omeprazole/esomeprazole/dexlansoprazole > pantoprazole). This order broadly reflects the acidity of the benzimidazole moiety. The higher the p*K*_a_ of the precursor, the more rapid is its conversion to the active molecule, and this can be expected to be true with both Brønsted and Lewis acids.

This is not the first study to look for PPI target proteins beyond the proton pump and the highly acidic parietal canaliculus. It can be assumed that PPIs can also be activated in acidic tissue micro‐environments (like tumour lesions and bone resorption sites), and more broadly in acidic subcellular compartments, that is, vesicles of the endolysosomal system. Indeed, lansoprazole was found to covalently bind and inhibit lysosomal cysteine proteases^18^, and the same may apply to omeprazole and esomeprazole^19^. It has been less clear if PPIs can be activated and conjugated to proteins in the cytosolic and nuclear environments of intact cells. Interestingly, it has been reported that PPIs inhibit the cytosolic enzyme dimethylarginine dimethylaminohydrolase (DDAH1)^20^ by forming a disulfide bond with the active site cysteine^21^. This cysteine coordinates a zinc ion in the enzyme’s resting state^22^. In light of our findings, it seems possible that the zinc ion of DDAH1 facilitates the reaction with PPIs.

Among our identified cytosolic/nuclear target proteins, C4 zinc-binding sites predominantly belong to the LIM, RING or PHD domains. Given the abundance of these domains, it is not obvious why the specific proteins identified in this study stand out as the most prominent targets of rabeprazole. However, PPI reactivity of C4 zinc clusters depends on several contextual factors: (1) overall accessibility; (2) the disposition of a cysteine ligand to dissociate; (3) the influence of side chains surrounding the zinc cluster; and (4) the stability of the resulting disulfide conjugate. Apparently, the observed target proteins are those that meet these conditions (cluster accessibility, zinc coordinability, drug binding affinity and conjugate stability).

Several of the reported side effects of long-term PPI use, including chronic kidney disease, cardiovascular disease and recurrent infections, may have a component of inflammatory dysfunction. It is therefore interesting to note that we identified all three members of the protein family that binds interferon regulatory factor 2 as targets of rabeprazole. These appear to have diverse functions in relation to infection and immunity^23^. The emerging question of whether these or other non-canonical target proteins of rabeprazole are connected to adverse medical side effects remains to be addressed in future research.

## Methods

### Preparation of cell lysates for bioorthogonal labelling of Rabazi target proteins

HEK293 MSR or PACO17 cells were grown to 70–80% confluency in culture medium lacking geneticin. Following aspiration of the medium, cells were washed once with Dulbecco’s Balanced Salt Solution (Gibco catalogue no. 14190094). A ×1,000 stock solution of Rabazi or rabeprazole was prepared in dimethyl sulfoxide (DMSO) solvent and diluted 1:1,000 into labelling medium (FluoroBrite DMEM, 1% foetal bovine serum (FBS)). The labelling solutions were vortexed, checked for precipitates and immediately added to the cells. The cells were incubated with the labelling medium at 37 °C and 5% CO_2_ for 90 min (unless otherwise indicated). The labelling medium was aspirated, and the cells were incubated on ice with 100 mM NEM (Sigma-Aldrich) in Dulbecco’s phosphate-buffered saline (DPBS) solution for 10 min. This step served to alkylate free thiols, which can react with dibenzocyclooctyne (DBCO) and thereby create non-specific labelling^24^. The NEM solution was removed and the required volume (60 mm, 50 µl; 100 mm, 100 µl; 150 mm, 200 µl) of 1% Triton cell lysis buffer (Cell Signaling catalogue no. 9803) supplemented with protease inhibitor mix G (Serva Electrophoresis) was added. Lysed cells were collected with a cell scraper and frozen in liquid nitrogen. After thawing on ice, lysates were sonicated three times for 1 min each in an ultrasound bath, with 2 min incubation on ice between cycles. The lysates were cleared from the insoluble material by centrifugation at 13,300*g* for 10 min at 4 °C. The supernatant was collected and centrifugation repeated once. The protein concentration of the final lysate was determined using the Bradford method (Bio-Rad Protein Assay).

### Labelling of Rabazi target proteins with DBCO–Cy5

To attach a fluorescent label to protein-conjugated Rabazi, strain-promoted alkyne–azide cycloaddition of DBCO–Cy5 (Sigma-Aldrich) was carried out. Cell lysates were diluted with DPBS to a final protein concentration of 1 µg µl^–1^ and then incubated with DBCO–Cy5 at a final concentration of 1 µM for 2 h at r.t. Subsequently, proteins were precipitated through methanol–chloroform precipitation as described by Wessel and Flügge^25^. Protein pellets were dissolved in Laemmli sample buffer (11.7% (v/v) glycerol, 1.17% SDS, 25 mM Tris, 0.12% bromophenol blue; pH 6.8) without reducing agents. Cy5-labelled proteins were resolved by SDS-PAGE analysis and Cy5 fluorescence was detected using the 700 nm channel of a LI-COR Odyssey Fc imager. For the data shown in Supplementary Fig. 1c–g, labelled proteins were transferred onto a nitrocellulose membrane by wet transfer and protein transfer was verified by Ponceau-S staining before detection of Cy5 fluorescence.

### Enrichment of Rabazi target proteins on DBCO–agarose

The protocol for enrichment and MS analysis of azide-labelled proteins was modified from the literature^26^. DBCO–agarose beads (Click Chemistry Tools) were equilibrated in PBS containing 0.5% NP-40 detergent. Cell lysates were diluted with DPBS to a final protein concentration of 2 µg µl^–1^ in 1.5 ml and incubated with 40 µl (50:50 slurry) DBCO–agarose beads by overhead rotation for 18 h at r.t. The beads were washed three times with a wash buffer (100 mM Tris, 1% SDS, 250 mM NaCl, 5 mM EDTA; pH 8.0), then three times with DPBS containing 1% SDS and finally three times with DPBS. Disulfide-bound proteins were eluted by reduction in 40 μl elution buffer (100 mM Tris, pH 8.8; 10 mM (tris(2-carboxyethyl)phosphine (TCEP), 1% SDS) for 2 h at 37 °C and 500 rpm.

### Identification of Rabazi target proteins by MS

Some 40 µl of each sample were subjected to an in-solution tryptic digest using a modified version of the single-pot, solid-phase-enhanced sample preparation (SP3) protocol^27,28^. Lysates were added to Sera-Mag Beads (Thermo Scientific, nos 4515-2105-050250 and 6515-2105-050250) in 10 µl of 15% formic acid and 30 µl of ethanol. Binding of proteins was achieved by shaking for 15 min at r.t. SDS was removed by four subsequent washes with 200 µl of 70% ethanol. Proteins were digested overnight at r.t. with 0.4 µg of sequencing grade modified trypsin (Promega, no. V5111) in 40 µl HEPES buffer/NaOH, pH 8.4 in the presence of 1.25 mM TCEP and 5 mM chloroacetamide (Sigma-Aldrich, no. C0267). Beads were separated and washed with 10 µl of an aqueous solution of 2% DMSO, and the combined eluates were dried down. Peptides were reconstituted in 10 µl of H_2_O and reacted for 1 h at r.t. with 80 µg of TMT10plex label reagent (Thermo Scientific, no. 90111) dissolved in 4 µl of acetonitrile (ACN)^29^. Each replicate was analysed in a separate tandem mass tag (TMT) experiment (*n* = 3). Samples were labelled in the following order: drug without azide in increasing concentrations (0.1 µM (TMT126), 1 µM (TMT127L) and 10 µM (TMT127H)), followed by the azide-containing drug in increasing concentrations (0.1 µM (TMT128L), 1 µM (TMT128H) and 10 µM (TMT129L)). Excess TMT reagent was quenched by the addition of 4 µl of an aqueous 5% hydroxylamine solution (Sigma, 438227). TMT-labelled and quenched peptides were dried in a SpeedVac and subsequently reconstituted in 100 µl of 0.1% formic acid. Peptides were pooled by mixing 5% (corresponding to 5 µl) of each sample together. The peptide pool was purified by reverse phase HPLC (OASIS HLB 96-well µElution Plate, Waters no. 186001828BA) and then analysed by LC-MS/MS on an Orbitrap Fusion Lumos mass spectrometer (Thermo Scientific) as described previously^30^. Peptides were separated using an UltiMate 3000 RSLCnano system (Dionex) equipped with a trapping cartridge (precolumn; C18 column PepMap 100, 5 mm, 300 μm inner diameter, 5 μm, 100 Å) and an analytical column (Acclaim C18 column PepMap 100; 75 μm × 50 cm, 3 mm, 100 Å) connected to a Nanospray Flex ion source. The peptides were loaded onto the trap column at 30 µl min^–1^ using solvent A (0.1% formic acid) and eluted using a gradient from 2% to 38% solvent B (0.1% formic acid in ACN) over 82 min and then to 80% at 0.3 µl min^–1^ (all solvents were of LC-MS grade). The Orbitrap Fusion Lumos was operated in positive ion mode with a spray voltage of 2.4 kV and capillary temperature of 275 °C. Full-scan MS spectra with a mass-to-charge ratio (m/z) range of 375–1,500 were acquired in profile mode using a resolution of 120,000 with a maximum injection time of 50 ms, automatic gain control (AGC) operated in standard mode and a radio frequency (RF) lens setting of 30%. Fragmentation was triggered for 3 s cycle time for peptide-like features with charge states of 2–7 on the MS scan (data-dependent acquisition). Precursors were isolated using the quadrupole with a window of 0.7*m*/*z* and fragmented with a higher-energy collisional dissociation (HCD) collision energy of 36%. Fragment mass spectra were acquired in profile mode and a resolution of 30,000. Maximum injection time was set to 94 ms or a normalized AGC target of 200%. The dynamic exclusion was set to 60 s. Acquired data were analysed using IsobarQuant^31^ and Mascot v.2.4 (Matrix Science) using a reverse UniProt FASTA *Homo sapiens* database (UP000005640 from May 2016) including common contaminants. The following modifications were taken into account: carbamidomethyl (C, fixed), TMT10plex (K, fixed), acetyl (N-term, variable), oxidation (M, variable) and TMT10plex (N-term, variable). The mass error tolerance for full-scan MS spectra was set to 10 ppm and for MS/MS spectra to 0.02 Da. A maximum of two missed cleavages were allowed. A minimum of two unique peptides with a peptide length of at least seven amino acids and a false discovery rate below 0.01 were required on the peptide and protein level^32^.

The raw output files of IsobarQuant (protein.txt files) were processed using the R programming language. Contaminants were filtered out and only proteins that were quantified with at least two unique peptides were considered for the analysis. Moreover, only proteins that were identified in two out of three MS runs were kept. Some 437 proteins passed the quality control filters. The log2-transformed raw TMT reporter ion intensities (‘signal_sum’ columns) were first cleaned for batch effects using limma^33^ and further normalized using vsn (variance stabilization normalization)^34^. Missing values were imputed with the knn method using the MSnbase package^35^. Proteins were tested for differential expression using the limma package. The replicate information was added as a factor in the design matrix given as an argument to the ‘lmFit’ function of limma. Also, imputed values were given a weight of 0.05 in the ‘lmFit’ function. A protein was annotated as a hit with a false discovery rate smaller than 5% and a fold change of at least 50%, and as a candidate with a false discovery rate below 20% and a fold change of at least 50%.

### Affinity purification of SBP-tagged DENR

HEK293 MSR cells were seeded in 150 mm dishes, transfected with pcDNA3-SBP-DENR (and corresponding constructs encoding DENR mutants; molecular mass of WT SBP-DENR, 26.7 kDa; SBP-DENR*, 26.6 kDa) using Lipofectamine 2000 (Invitrogen) according to the manufacturer’s protocol. After 48 h, transfected cells were treated with 10 µM Rabazi or 10 µM Rabazi-ΔO in FluoroBrite (Gibco) with 1% FBS for 15 min at 37 °C. Following medium removal, cells were treated on ice with 100 mM NEM in PBS for 10 min. Cells were then lysed with 1% Triton-X in tris-buffered saline (TBS) for 30 min at 4 °C. Cell lysates were cleared by centrifugation at 16,000*g* for 30 min at 4 °C. Streptavidin sepharose beads (Cytiva), equilibrated with TBS, were added to the cleared cell lysate. The slurry was rotated at 4 °C for 1–2 h. Beads were washed two times with 0.1% Triton-X in TBS and then treated with 100 mM NEM in TBS for 10 min at r.t. Finally, beads were incubated with 1 µM DBCO–Cy5 for 45 min at r.t. with protection from light. Beads were washed two times with 0.1% Triton-X in TBS. SBP-tagged DENR was eluted by incubation with SDS sample buffer at 95 °C for 5 min. Eluates were analysed by SDS-PAGE. Cy5-conjugated proteins were visualized using the 700 nm channel of a LI-COR Odyssey XF imager. Protein loading was visualized by Coomassie staining (InstantBlue, Abcam).

### Labelling of recombinant DENR with Rabazi–DBCO–Cy5

‘Pre-clicked’ Rabazi–DBCO–Cy5 (10) and Rabazi-ΔO–DBCO–Cy5 were prepared by reacting Rabazi or Rabazi-ΔO with DBCO–Cy5. Equal volumes of Rabazi or Rabazi-ΔO (10 mM in DMSO) and DBCO–Cy5 (10 mM in DMSO) were incubated for 3–5 h at r.t., protected from light. The resulting product solution (5 mM) was diluted in DMSO to a final concentration of 300 µM, then aliquoted and stored at –20 °C. Purified recombinant DENR–MCTS1 was diluted in reaction buffer (50 mM Tris, 150 mM NaCl, pH 7.5) to a final concentration of 0.2 µg µl^–1^. NEM was diluted in reaction buffer to a final concentration of 40 mM. Some 10 µl of protein dilution and 10 µl of NEM dilution were mixed and incubated for 10 min at r.t. Rabazi–DBCO–Cy5 or Rabazi-ΔO–DBCO–Cy5 stock solutions were diluted in reaction buffer to a final concentration of 3 µM, and 10 µl was added to the DENR–MCTS1/NEM mixture. The labelling reaction was incubated for 2.5 min at r.t. (unless otherwise specified) before adding ×6 SDS sample buffer. Samples were denatured at 95 °C for 10 min and separated by 15% SDS-PAGE. Cy5-conjugated proteins were visualized using the 700 nm channel of a LI-COR Odyssey Fc imager. Protein loading was visualized by Coomassie staining (InstantBlue, Abcam).

For the experiment shown in Fig. 3a, DENR (0.2 µg µl^–1^) was incubated with Rabazi (2 µM) and various concentrations of NEM (0, 1, 10, 50, 100 mM) for 10 min at r.t. The incubation with 100 mM NEM was additionally performed in the presence of 0.1% SDS, and incubated for 10 min at 72 °C. Subsequently, samples were reacted with DBCO–Cy5 (1 µM) for 2 h at r.t., in the dark. All samples were analysed by SDS-PAGE. For the experiment shown in Fig. 3b, recombinant DENR (10 µM) was incubated with Rabazi (30 µM) and NEM (20 mM), in the absence or presence of EDTA (2.5 mM), TPEN (2.5 mM) or SDS (1%). After 5 min at r.t., the protein was precipitated by methanol–chloroform extraction, as described previously^25^. The protein pellets were resuspended in 20 µl of 100 mM NEM, 50 mM Tris and 150 mM NaCl. Each sample was incubated with DBCO–Cy5 (1 µM) for 2 h at r.t., in the dark. All samples were analysed by SDS-PAGE.

### Zinc quantification using metallochromic indicators

To determine the concentration of zinc in a protein sample, the metallochromic indicator PAR was used. To release zinc from the protein, the protein solution (20 µM) was mixed with an equal volume of a solution of 8 M guanidinium chloride and 100 mM NEM in double distilled H_2_O. Per well of a 96-well plate, 25 µl of PAR solution (400 µM in 50 mM HEPES, pH 7.4) was added to 75 µl of the denatured and alkylated protein sample. After incubation for 10 min at r.t., the absorption of the Zn–PAR complex was measured at 494 nm, using a spectrophotometer (Pherastar, BMG). To obtain zinc concentrations, a standard curve of ZnCl_2_ was prepared. Zinc released from DENR by NEM or PPIs was also measured using PAR. For zinc release assays, the protein was diluted to a final concentration of 7.5 µM in 50 mM Tris–HCl, 150 mM NaCl, pH 7.5. PAR was added to a final concentration of 100 µM along with NEM (750 µM) or PPI (375 µM). For the zinc release assay shown in Supplementary Fig. 3b, 100 µM Zincon (Sigma-Aldrich) was used instead of PAR and the absorption of the Zn–Zincon complex was measured at 618 nm. To control for protein precipitation, measurements were background corrected against samples without Zincon.

### Zinc quantification by inductively coupled plasma optical emission spectroscopy

For inductively coupled plasma optical emission spectroscopy (ICP-OES) measurements, purified protein was subjected to two steps of buffer exchange (50 mM sodium phosphate) using PD10 desalting columns (Cytiva). The protein concentration of WT DENR was determined by measuring absorption at 280 nm with a NanoDrop spectrophotometer and using the extinction coefficient of the DENR–MCTS1 heterodimeric complex as calculated with the Expasy ProtParam tool. To control for variations in DENR–MCTS1 stoichiometry, the concentration of DENR mutants was determined by quantifying Coomassie-stained bands relative to WT DENR. ICP-OES samples were subjected to open acid digestion prior to measurement. To this end, 1 ml of sample solution was incubated with 2 ml of sub-boiling distilled nitric acid (65%), for 1 h at 90 °C. Following the addition of Milli-Q water to a total volume of 12 ml, samples were analysed with an ICP-OES instrument (Agilent 720). A long torch in combination with a Conical nebulizer and a cyclone chamber was used for sample introduction. Zinc was measured at a wavelength of 213.857 nm, using certified reference materials SPS-SW1 and SPS-SW2, adjusted with an equivalent acid concentration. The calculated recoveries were between 96% and 101%. Measuring two samples in triplicate each, the root mean square deviations were below 0.5%.

### Intact protein MS

For intact protein MS, 5 µM recombinant DENR–MCTS1 was incubated with different concentrations of NEM (Extended Data Fig. 3b) or 25 µM rabeprazole (Extended Data Figs. 2a and 4a) in 50 mM Tris–HCl, 150 mM NaCl and 10% DMSO for 15 min at r.t. Samples were then either snap-frozen in liquid nitrogen and stored at –20 °C before analysis, or immediately injected to the mass spectrometer (Extended Data Fig. 4a). The intact masses of proteins were determined via electrospray ionization MS using a quadrupole time-of-flight mass spectrometer (maXis, Bruker). Protein samples, with a concentration of 0.25 µM, underwent desalting by HPLC (Agilent). Two hundred microlitres of the protein samples were loaded onto a reversed-phase trapping column (0.8 × 2 mm; Poros R1, Applied Biosystems). Following a 3 min wash with 0.3% formic acid at a flow rate of 300 μl min^–1^, proteins were eluted with a solution of 40% isopropanol, 5% ACN and 0.3% formic acid at a flow rate of 40 μl min^–1^. Protein mass spectra obtained were deconvoluted using the MaxEnt algorithm, implemented in Compass DataAnalysis 4.2 software (Bruker).

### Analysis of thiol modifications by differential alkylation

To identify the preferred target site of rabeprazole, 69 µg of purified His-DENR*–MCTS1 was diluted to 5 µM in 50 mM Tris–HCl and 150 mM NaCl, pH 7.5, and 10% DMSO and incubated with five-fold molar excess rabeprazole for 15 min at r.t. To stop the reaction and remove excess rabeprazole, the modified protein was precipitated through chloroform–methanol precipitation as described in the literature^25^. Samples were subjected to an in-solution tryptic digest using a modified version of the SP3 protocol^27,28^. After vortexing, 30 µl of Sera-Mag A beads, 30 µl of Sera-Mag B beads and 160 µl of distilled H_2_O were combined in a low-binding Eppendorf tube. The tube was placed on a magnetic rack, allowing the beads to settle for about 2 min. Following the removal of the supernatant, the beads were rinsed three times with 200 µl of distilled H_2_O. The beads were then stored in 30 µl of H_2_O in the fridge at 4 °C. The modified protein was resuspended in 100 mM TEAB buffer and split into equal aliquots. Three replicates were treated with the reduction/alkylation buffer (100 mM TEAB pH 8.5, 1% SDS, 10 mM TCEP, 40 mM chloroacetamide), and the other three were treated with the alkylation buffer (100 mM TEAB pH 8.5, 1% SDS, 40 mM chloroacetamide). Both sets were incubated at 95 °C for 5 min and then at 70 °C for 25 min. After cooling to r.t., ACN was added to a final concentration of 60%. Each sample was mixed with 2 µl of pre-rinsed beads (as described above), thoroughly mixed and incubated for 20 min on a shaking incubator. Subsequently, the samples were briefly centrifuged and placed on a magnetic rack, and the supernatant was carefully removed. Beads were washed with 200 µl of wash buffer (60% v/v ACN, 40% v/v 100 mM TEAB) by sonication in an ultrasonic bath for 7 min and incubating for an additional 20 min in a shaking incubator. After a brief centrifugation, the supernatant was removed. Beads were washed twice with 200 µl of 80% ethanol, washed once with 180 µl of 100% ACN and finally air dried. The dried beads were reconstituted in 100 µl of 100 mM TEAB, pH 8.5, by sonication for 7 min and quickly spinning down the vials. Lys-C was added at a 1:50 enzyme/protein ratio, and the solution was mixed and incubated for 4 h at 37 °C. Next, trypsin was added in a 1:50 ratio, followed by overnight incubation at 37 °C. The samples were acidified with trifluoroacetic acid (TFA) to below pH 2, and the supernatant was transferred to fresh vials. Desalting, reduction and dimethyl labelling of the peptides were performed using self-assembled C18 Empore extraction disc StageTips (3M)^36^. Each StageTip was conditioned with MeOH followed by 80% ACN/0.1% TFA, equilibrated with 0.1% TFA and loaded with acidified peptides. The set of three replicates treated with alkylation buffer in the previous step was flushed with 300 µl of 10 mM TCEP for at least 30 min, followed by a wash with 0.1% TFA. The set of three replicates treated with the reduction/alkylation buffer in the previous step was flushed with 50 mM TEAB, followed by a wash with 0.1% TFA. Finally, replicates treated with the reduction/alkylation buffer and replicates treated with the alkylation buffer were flushed for 10 min with 300 µl of light labelling solution (270 µl 100 mM TEAB of pH 8.0, 15 µl 4% formaldehyde, 15 µl 0.6 M cyanoborohydride) and 300 µl heavy labelling solution (270 µl 100 mM TEAB of pH 8.0, 15 µl 4% deuterated and ^13^C-labelled formaldehyde, 15 µl 0.6 M deuterated cyanoborohydride), respectively. The toxic flow-through was discarded, and the StageTip was transferred into a fresh microcentrifuge tube. Following washing with 0.1% TFA, peptides were eluted using 80% ACN/0.1% TFA. The elution step was repeated once, and light and heavy labelled peptides were mixed in a 1:1 ratio. Samples were suspended in 0.1% TFA, and an equivalent of 1 µg of peptides was analysed using an UltiMate 3000 LC system coupled to an Orbitrap Q Exactive HF (Thermo Fisher). An in-house-packed analytical column (75 µm × 200 mm, 1.9 µm ReproSil-Pur 120 C18-AQ material; Dr. Maisch) was used. Mobile phase solutions were prepared as follows: solvent A was 0.1% formic acid and 1% ACN, and solvent B was 0.1% formic acid and 89.9% ACN. Peptides were separated in a 60 min linear gradient starting from 3% B and increased to 23% B over 50 min and to 38% B over 10 min, followed by wash-out with 95% B. The mass spectrometer was operated in data-dependent acquisition mode, automatically switching between MS and MS2. MS spectra (*m*/*z*, 400–1,600) were acquired in the Orbitrap at 60,000 (*m*/*z*, 400) resolution, and MS2 spectra were generated for up to 15 precursors with a normalized collision energy of 27 and an isolation width of 1.4*m*/*z*. The MS/MS spectra were searched against the modified protein sequence and a customized contaminant database (part of MaxQuant, MPI Martinsried) using Proteome Discoverer 2.5 with Sequest HT (Thermo Fisher Scientific). The fragment ion mass tolerance was set to 0.02 Da, and the parent ion mass tolerance was set to 10 ppm. Trypsin was specified as the enzyme. Dimethylation was set as a fixed modification of lysine and the N-terminal end of peptides. The following modifications were set as dynamic: oxidation (methionine) and carbamidomethylation (cysteine). Peptide quantification was done using the precursor ion quantifier node with the Top N Average (*n* = 3) method set for protein abundance calculation.

### Identification of the rabeprazole conjugation site using LC-MS/MS

To identify the site of rabeprazole modification, 10 µg of purified His-DENR–MCTS1 per sample was diluted to 10 µM in 50 mM Tris–HCl, 150 mM NaCl, pH 7.5 and incubated with a five-fold molar excess of rabeprazole and 30 mM iodoacetamide. After 15 min of incubation at r.t., SDS was added to a final concentration of 1% to denature the protein and promote full alkylation of unmodified thiols. Proteins were digested as described in the previous section, but without using reducing or alkylating agents prior to digestion. Following digestion, samples were desalted with self-assembled C18 Empore StageTips (3M) following the protocol in the literature^36^. The peptides were then dried in a centrifugal evaporator and resuspended in 0.1% TFA. An equivalent of 2 µg of peptides was analysed using an UltiMate 3000 LC system coupled to an Orbitrap Q Exactive HF (Thermo Scientific) with minor modifications to the method described in the section above. Due to the lower sample complexity, peptides were separated using a 25 min linear gradient, starting from 3% B, increasing to 23% B over 21 min and then to 38% B over 4 min, followed by a wash-out at 95% B. MS/MS spectra were searched against the WT DENR sequence and a customized contaminant database (part of MaxQuant, MPI Martinsried) using Proteome Discoverer 2.5 with Sequest HT (Thermo Fisher Scientific). The fragment ion mass tolerance was set to 0.02 Da, and the parent ion mass tolerance, to 10 ppm. Trypsin was specified as the digestion enzyme. Dynamic modifications included cysteine modification by rabeprazole (+342.128 Da) and its hydrolysed form (+225.082 Da), along with oxidation (methionine), deamidation (asparagine and glutamine) and carbamidomethylation (cysteine). The protein N-terminal modifications allowed were acetylation, methionine loss and combined methionine loss with acetylation. Peptide quantification was performed using the precursor ion quantifier node with the Top N Average (*n* = 3) method for protein abundance calculation.

### NMR spectroscopy

NMR experiments were collected on a Bruker Avance III 700 MHz spectrometer at 298 K (25 °C) with samples containing 10% D_2_O as a locking agent. The ^15^N-labelled DENR^26–98^–MCTS1 was concentrated to 140 μM using Amicon Ultra-15 10 kDa molecular weight cut-off centrifugal filters and prepared in a sample buffer consisting of 20 mM Tris (pH 7.5) and 100 mM NaCl. Rabeprazole and rabeprazole-ΔO were dissolved in DMSO at a concentration of 100 mM to avoid dilution effects during the experiment. Changes in the chemical shifts of the small-molecule compounds and ^15^N-labelled DENR^26–98^–MCTS1 were monitored over time after addition of a five-fold molar excess of the drug, by collecting Carr–Purcell–Meiboom–Gill 1D ^1^H and 2D ^1^H–^15^N HSQC experiments with apodization weighted sampling, respectively^37^. The Carr–Purcell–Meiboom–Gill pulse train in the 1D experiment filters broader signals originating from protons associated with macromolecules. The raw NMR data were processed and analysed using NMRpipe and NMRFAM-Sparky^38,39^. As controls, changes in the chemical shifts of the small molecules were monitored as well, in samples lacking DENR but otherwise identical.

### Molecular modelling

To model the interface between the DENR–MCTS1 complex (PDB no. 6MS4) and rabeprazole, we first prepared the protein structure by performing an energy minimization with restrained Cα atoms. The C terminus must undergo substantial conformational changes to open the binding site to rabeprazole. To account for this opening, we initially truncated the DENR C terminus after A51 to expose the zinc-binding site (how we restored the full protein is detailed below). A rabeprazole structure was generated from the Simplified Molecular Input Line Entry System (SMILES) and minimized to obtain (*R*)-rabeprazole, followed by inverting atoms at the chiral centre to obtain (*S*)-rabeprazole. Both protein and ligand structures were then taken to perform docking using AutoDock Vina^40^ for zinc metalloproteins^41^. A cubic grid with a spacing of 0.375 Å and 40 grid points along each axis was used with the exhaustiveness set to a value of 32. For every rabeprazole enantiomer, two differently oriented docked configurations with a zinc-coordinated benzimidazole nitrogen and high docking scores were chosen for further investigation. Next, the truncated DENR C terminus was added to the docked structures to restore the full protein environment at the docking site. Steric clashes between the DENR C terminus and rabeprazole were resolved by opening the C-terminal lid along the D49 *φ* dihedral angle, followed by energy minimization. For every DENR–MCTS1–rabeprazole complex, three technical replicates were equilibrated and simulated for at least 400 ns to sample stable binding poses. Minimizations involving protein structures and MD simulations were performed using GROMACS^42^ v.2023.4 with the amber force fields ff14SB^43^ for the protein complex, GAFF2 (ref. ^44^) for rabeprazole and ZAFF^45^ for the zinc cluster. Simulations were performed with 2 fs time steps at 300 K and 1 bar.

### Reporting summary

Further information on research design is available in the Nature Portfolio Reporting Summary linked to this article.

## Online content

Any methods, additional references, Nature Portfolio reporting summaries, source data, extended data, supplementary information, acknowledgements, peer review information; details of author contributions and competing interests; and statements of data and code availability are available at 10.1038/s41557-025-01745-8.

## Supplementary information

Supplementary Information Supplementary Figs. 1–3, Methods and Synthesis notes.

Reporting Summary

Supplementary Table 1 Significantly enriched rabeprazole target proteins.

Supplementary Data 1 Numerical source data for Supplementary Fig. 2.

Supplementary Data 2 Numerical source data for Supplementary Fig. 3.

## Source data

Source Data Fig. 1 Numerical source data.

Source Data Fig. 2 Unprocessed Coomassie gels and numerical source data.

Source Data Fig. 3 Unprocessed Coomassie gels and numerical source data.

Source Data Fig. 4 Numerical source data.

Source Data Fig. 5 Numerical source data.

Source Data Fig. 6 Numerical source data.

Source Data Extended Data Fig. 2 Numerical source data.

Source Data Extended Data Fig. 3 Numerical source data.

Source Data Extended Data Fig. 4 Numerical source data.

Source Data Extended Data Fig. 5 Numerical source data.

## Data Availability

All data generated and analysed in this study are included in this Article. The mass spectrometry proteomics data have been deposited to the ProteomeXchange Consortium via the PRIDE partner repository with the dataset identifier PXD048457. Source data are provided with this paper.
