# Supplementary material for: Site-specific activation of the proton pump inhibitor rabeprazole by tetrathiolate zinc centres
Source: Nat Chem. 2025 Feb 20;17(4):507–17. doi: 10.1038/s41557-025-01745-8 (PMC11964933; doi:10.1038/s41557-025-01745-8)

nature chemistry

Supplementary information

https://doi.org/10.1038/s41557-025-01745-8

# Site-specific activation of the proton pump inhibitor rabeprazole by tetrathiolate zinc centres

In the format provided by the authors and unedited

**Table of Contents**


Synthesis Notes 2

General information for chemical synthesis 2
Synthesis procedures 2
NMR Spectra 5

Supplementary Methods 11

Cell culture 11
Plasmid construction for mammalian DENR expression 11
Plasmid construction for bacterial DENR expression 11
Purification of recombinant DENR-MCTS 12

References 14

Supplementary Figures 15

Supplementary Figure S1 15
Supplementary Figure S2 17
Supplementary Figure S3 18
Uncropped blots for Supplementary Figures S1C-G 19

## Synthesis Notes

### General information for chemical synthesis

Chemicals and solvents were purchased from commercial sources at the highest level of purity and used without purification. All reactions were stirred magnetically and external bath temperatures are reported for heating. Thin layer chromatography (TLC) was carried out on glass silica plates (TLC Silica gel 60 $F_{254}$; Merck). TLC visualization was accomplished using 254 nm UV light, iodine-saturated silica gel ($I_2$), or permanganate ($KMnO_4$) stain. High resolution mass spectrometry was recorded on a Bruker ApexQe FT-ICR instrument (Department of Organic Chemistry, Heidelberg University). NMR spectra were recorded on a Bruker Avance III 9.4 T or a Bruker Avance 14.1 T operating at 400 MHz for $^1$H nuclei and 101 MHz for $^{13}$C nuclei, respectively. Spectra were recorded at 298.1 K and are referenced to residual solvent: $CHCl_3$ (7.26 ppm; 77.16 ppm); DMSO-$d_5$ (2.50 ppm; 39.52 ppm). All compounds were found to have ≥95% purity, controlled by analytical HPLC/UV/ELSD/MS and confirmed by $^1$H NMR and $^{13}$C NMR. Analytical HPLC was performed on an Agilent 1260 Infinity system equipped with a 6120 Quadrupole mass detector and evaporative light scattering detector (ELSD). *Column*: Kinetex 2.6 µm C18 100 Å, LC column 50 x 2.1 mm; 40 °C; flow rate: 0.06 mL/min. *Solvent system*: A: $H_2O$, 0.01% HCOOH, B: MeCN. *Gradient*: 1% B → 90% B over 6 min, then 90% B → 99% B over 2 min.

### Synthesis procedures

Note: Rabeprazole is sold as a sodium salt. We therefore prepared the sodium salt of Rabazi and used it in all experiments. Depictions of Rabazi in the figures show the free acid form for the sake of simplicity, but it is to be understood that Rabazi sodium was always used.

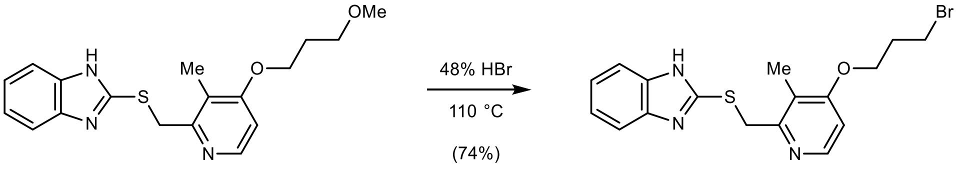


**Rabeprazole thioether** **S1**

**2-(((4-(3-bromopropoxy)-3-methylpyridin-2-yl)methyl)thio)-1H-benzo[d]imidazole•2HBr** (**S1**): A suspension of Rabeprazole thioether (2.000 g, 5.823 mmol) in 48% hydrobromic acid (50 mL) was heated to 110 °C. Within 30 min, the suspension became a yellow solution, and within 2.5 h, a white precipitate began to form. After 20 h, the reaction mixture was cooled to room temperature and then –20 °C. After 2 h, the precipitate was collected via vacuum filtration and washed with ice cold water (2 x 25 mL). The washing steps should be performed rapidly to avoid material loss as the product has appreciable solubility in water. The white solid was dried under high vacuum to give 2.378 g (74%) of the desired product as the bis HBr salt. An additional 512 mg (7%) can be obtained by partial concentration of the mother liquor followed by cooling, filtration, and washing: **TLC** (of the free base) $R_f$ 0.36 (5% MeOH in $CH_2Cl_2$; UV, $KMnO_4$, $I_2$); **$^1$H NMR** (400 MHz, DMSO-$d_6$) δ 8.68 (d, *J* = 6.8 Hz, 1H), 7.64 – 7.59 (m, 2H), 7.58 (d, *J* = 6.8 Hz, 1H), 7.38 – 7.31 (m, 2H), 4.88 (s, 2H), 4.43 (t, *J* = 5.8 Hz, 2H),

3.70 (t, $J$ = 6.5 Hz, 2H), 2.35 (app pent, $J$ = 6.1 Hz, 2H), 2.27 (s, 3H) ppm (NH peaks are in exchange with residual water in the spectrum). **LC/MS** (*m/z*): $[M+H]^+$ 392.0 and 394.0.

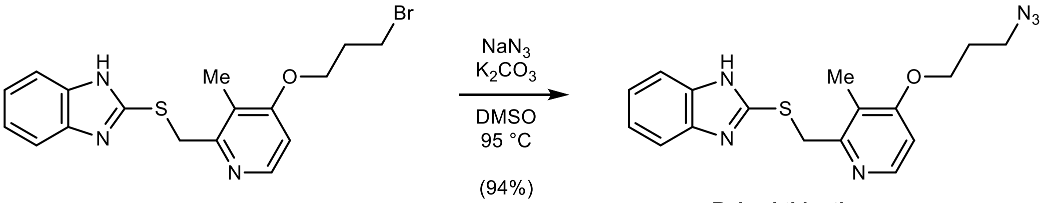


S1

Rabazi thioether
(RabaziΔO)

**2-(((4-(3-azidopropoxy)-3-methylpyridin-2-yl)methyl)thio)-1H-benzo[d]imidazole (Rabazi thioether or RabaziΔO)**: To a suspension of **S1** (2.358 g, 4.255 mmol, 1.0 equiv) and $K_2CO_3$(1.176 g, 8.51 mmol, 2.0 equiv) in DMSO (1 mL) was add a solution of $NaN_3$ in DMSO (17 mL, 0.5 M, 2.0 equiv) at room temperature. The mixture was submerged in a preheated 95 °C oil bath for 45 min, at which time LC/MS analysis showed consumption of the starting material. The reaction mixture was diluted with water (250 mL), brine (250 mL), and EtOAc (80 mL). The layers were separated and the aqueous layer was further extracted with EtOAc (3 x 80 mL). The combined organic layers were washed with brine (50 mL), dried ($MgSO_4$), filtered, and concentrated in vacuo. The residue was purified by automated flash chromatography (0 to 5% gradient of MeOH in $CH_2Cl_2$ with 0.5% $NH_4OH$ as pH modifier) to give 1.418 g (4.00 mmol, 94%) of the product as a sticky foam. **TLC** $R_f$ 0.26 (5% MeOH in $CH_2Cl_2$; UV, $KMnO_4$, $I_2$); **$^1$H NMR** (400 MHz, $CDCl_3$) δ 11.10 (br s, 1 H), 8.34 (d, $J$ = 5.8 Hz, 1H), 7.56 – 7.50 (m, 2H), 7.20 – 7.14 (m, 2H), 6.73 (d, $J$ = 5.8 Hz, 1H), 4.39 (s, 2H), 4.10 (t, $J$ = 5.9 Hz, 2H), 3.53 (t, $J$ = 6.5 Hz, 2H), 2.25 (s, 3H), 2.09 (app pent, $J$ = 6.2 Hz, 2H ppm); **$^{13}$C NMR** (101 MHz, $CDCl_3$) δ 164.0, 156.8, 151.7, 147.4, 137.6 (very br), 121.9, 121.2, 113.7 (very br), 106.1, 65.2, 48.1, 35.0, 28.6, 10.8 ppm (Note: benzimidazole tautomerism is responsible for the broad signals. In this compound, the four CH-bearing carbons on the benzimidazole appear as two peaks); **HRMS-ESI** (*m/z*): $[M+H]^+$ calcd for $C_{17}H_{19}N_6OS^+$: 355.1336; found: 355.1337.

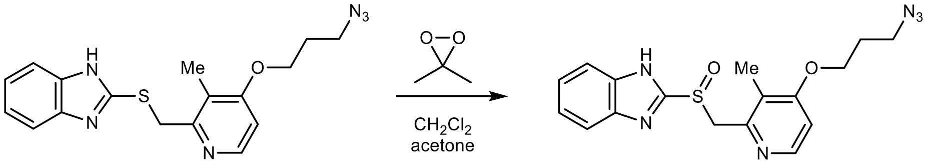


Rabazi thioeter
(RabaziΔO)

Rabazi

**2-(((4-(3-azidopropoxy)-3-methylpyridin-2-yl)methyl)sulfinyl)-1H-benzo[d]imidazole (Rabazi)**: To a solution of **Rabazi thioether** (210.8 mg, 0.595 mmol, 1.0 equiv) in $CH_2Cl_2$ (5 mL) was added a solution of dimethyldioxirane (11.9 mL, 0.05 M in acetone by iodometric titration, 1.0 equiv) dropwise at room temperature[1]. After 25 min, the reaction was deemed complete by TLC analysis. The reaction mixture was concentrated in vacuo and purified by column chromatography (5% MeOH in $CH_2Cl_2$) to give 147 mg (0.397 mmol, 66%) of the product as a grayish foam. Note: this reaction can also be conducted with *m*-CPBA as an oxidant with slightly lower yields. **TLC** $R_f$ 0.22 (5% MeOH in $CH_2Cl_2$; UV, $KMnO_4$, $I_2$); **$^1$H NMR** (400 MHz, $CDCl_3$) δ 8.29 (d, $J$ = 5.7 Hz, 1H), 8.00 – 7.35 (very br m, 2H),

7.31 – 7.26 (m, 2H), 6.68 (d, *J* = 5.7 Hz, 1H), 4.81 (d, *J* = 13.7 Hz, 1H), 4.74 (d, *J* = 13.7 Hz, 1H), 4.10 – 3.98 (m, 2H), 3.47 (t, *J* = 6.5 Hz, 2H), 2.12 (s, 3H), 2.04 (app pent, *J* = 6.2 Hz, 2H) ppm; **$^{13}$C NMR** (101 MHz, $CDCl_3$) δ 163.4, 153.3, 149.6, 148.5, 143.7 (very br), 134.5 (very br), 123.7 (very br), 123.0, 120.2 (very br), 112.2 (very br), 106.2, 65.0, 60.9, 48.1, 28.5, 11.2 ppm (Note: benzimidazole tautomerism is responsible for the broad signals. In this compound, the four CH-bearing carbons on the benzimidazole appear as four distinct peaks); **HRMS-ESI** (*m/z*): $[M+H]^+$ calcd for $C_{17}H_{19}N_6O_2S^+$: 371.1285; found: 371.1285.

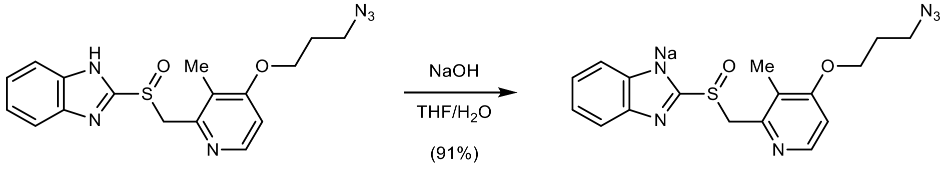


**Sodium 2-(((4-(3-azidopropoxy)-3-methylpyridin-2-yl)methyl)sulfinyl)benzo[d]imidazol-1-ide (Rabazi sodium salt)**: To a solution of **Rabazi** (130.1 mg, 0.351 mmol, 1.0 equiv) in THF (2.5 mL) was added a solution of NaOH (1.0 M, 251 µL, 0.351 mmol, 1.0 equiv) at room temperature. After 30 min, the mixture was concentrated in vacuo, dissolved in $H_2O$ (2.5 mL), frozen in liquid nitrogen, and lyophilized to give 125.9 mg (0.321 mmol, 91%) of an off-white powder: **$^1$H NMR** (400 MHz, DMSO-*d*$_6$) δ 8.30 (d, *J* = 5.7 Hz, 1H), 7.52 – 7.42 (m, 2H), 6.95 (d, *J* = 5.7 Hz, 1H), 6.92 – 6.86 (m, 2H), 4.68 (d, *J* = 13.0 Hz, 1H), 4.45 (d, *J* = 13.0 Hz, 1H), 4.12 (t, *J* = 6.0 Hz, 2H), 3.53 (t, *J* = 6.7 Hz, 2H), 2.18 (s, 3H), 2.02 (app pent, *J* = 6.4 Hz, 2H) ppm (Note: the sodium salt shows no line broadening as no benzimidazole tautomerism is possible); **$^{13}$C NMR** (101 MHz, DMSO-*d*$_6$) δ 162.5, 162.3, 152.4, 148.0, 146.6, 121.8, 118.3, 117.3, 106.0, 65.1, 60.0, 47.7, 27.9, 10.8 ppm; **HRMS-ESI** (*m/z*): $[M–H]^-$ calcd for $C_{17}H_{19}N_6O_2S^-$: 369.1132; found: 369.1137.

## NMR Spectra

1H NMR, 400 MHz, CDCl3, 298.1 K

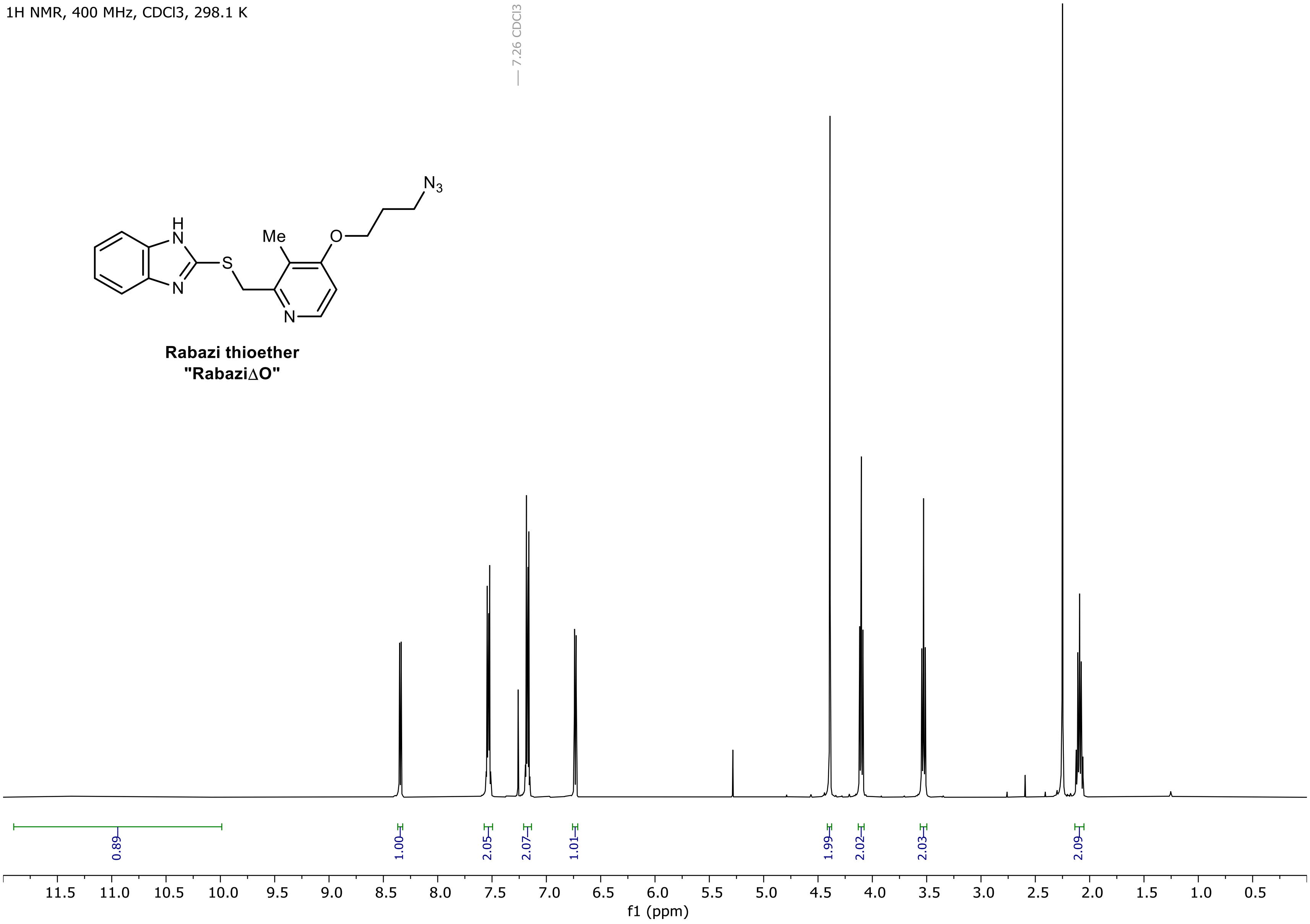


**Rabazi thioether**
**"RabaziΔO"**

13C NMR, 400 MHz, CDCl3, 298.1 K

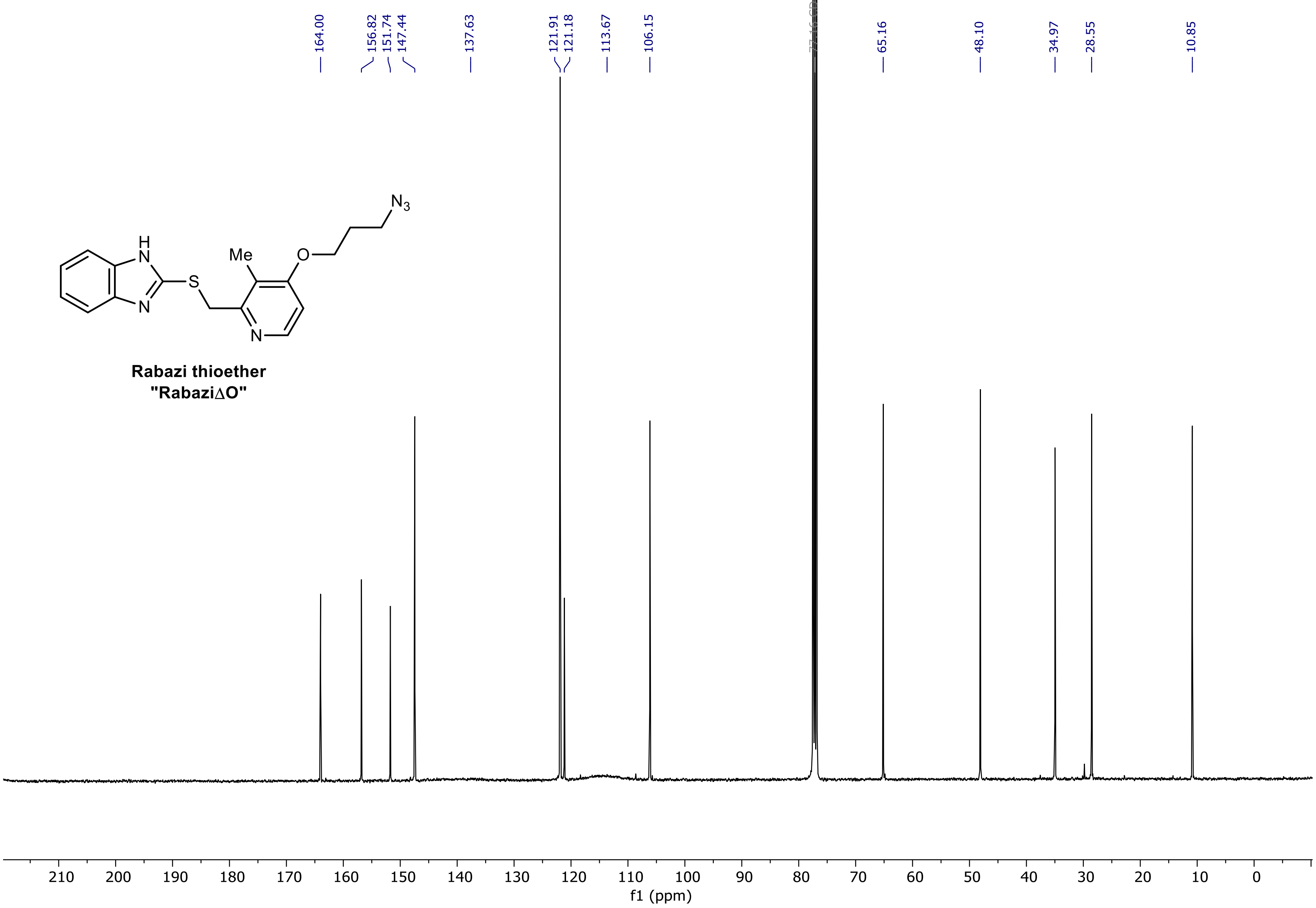


**Rabazi thioether**
**"RabaziΔO"**

1H NMR, 400 MHz, CDCl3, 298.1 K

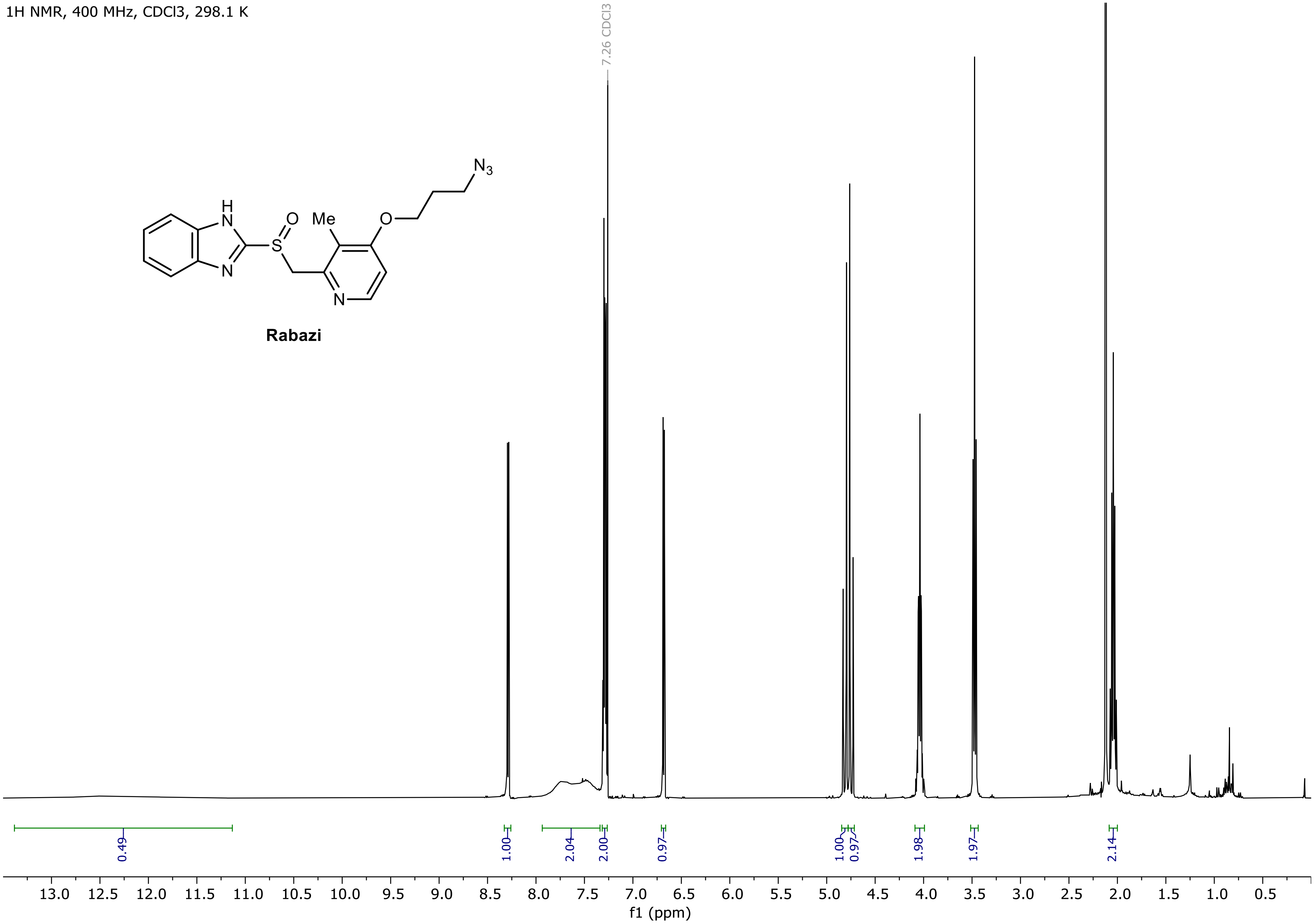

13C NMR, 100 MHz, CDCl3, 298.1 K, line broadening = 5 Hz

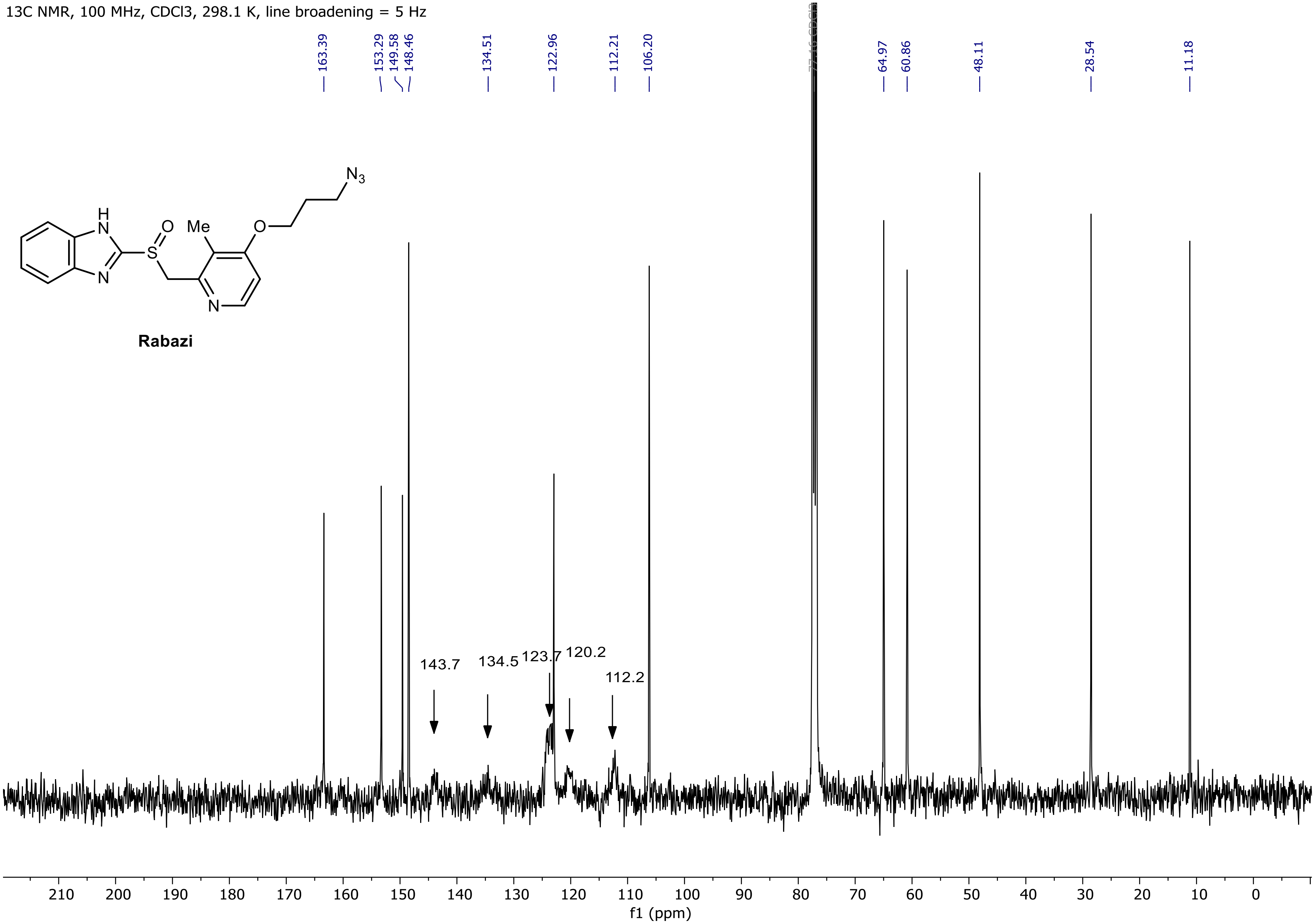

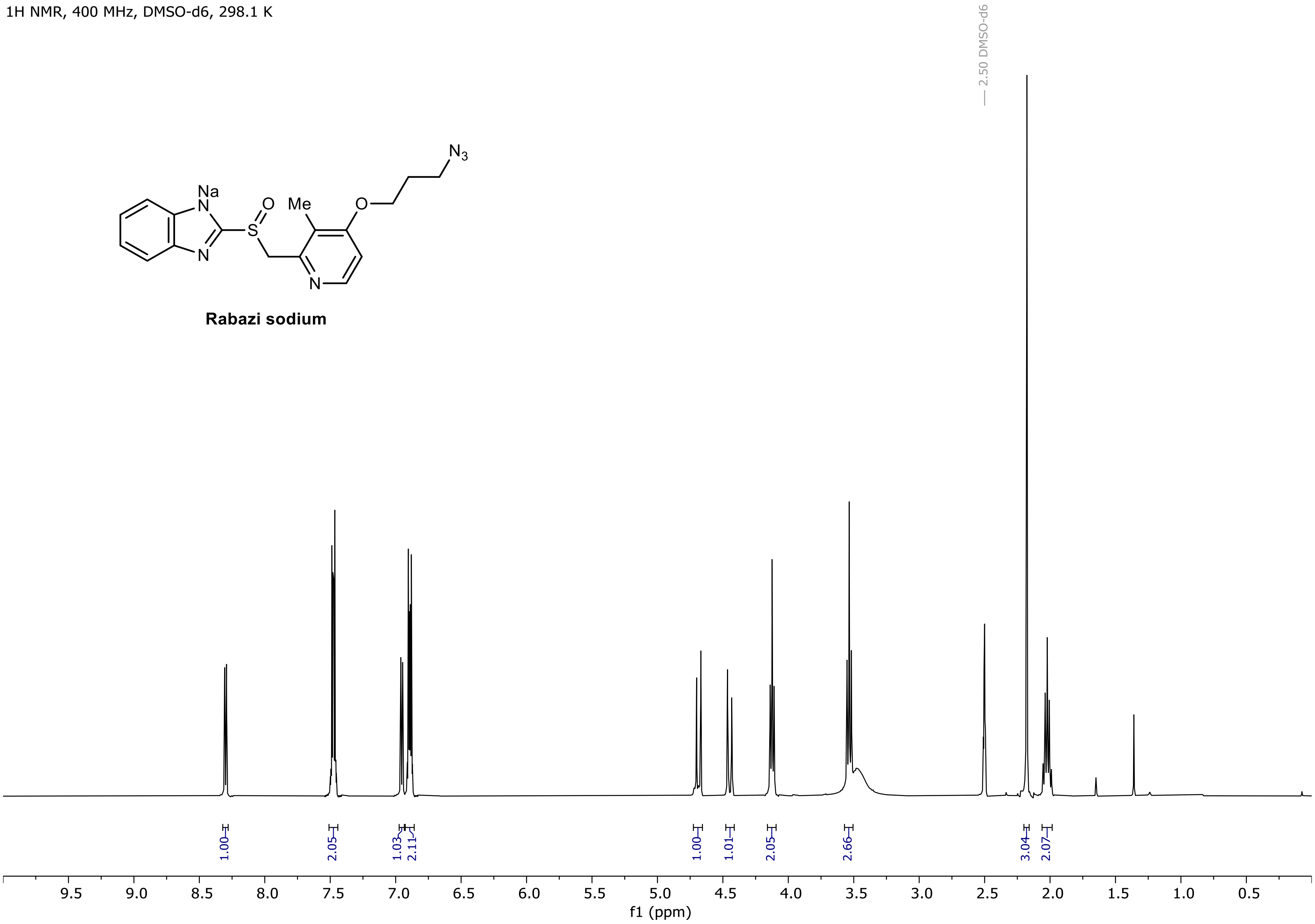
1H NMR, 400 MHz, DMSO-d6, 298.1 K
2.50 DMSO-d6
Na
N
N
S
O
Me
O
$N_3$
N
Rabazi sodium
1.00
2.05
1.03
2.11
1.00
1.01
2.05
2.66
3.04
2.07
9.5 9.0 8.5 8.0 7.5 7.0 6.5 6.0 5.5 5.0 4.5 4.0 3.5 3.0 2.5 2.0 1.5 1.0 0.5
f1 (ppm)

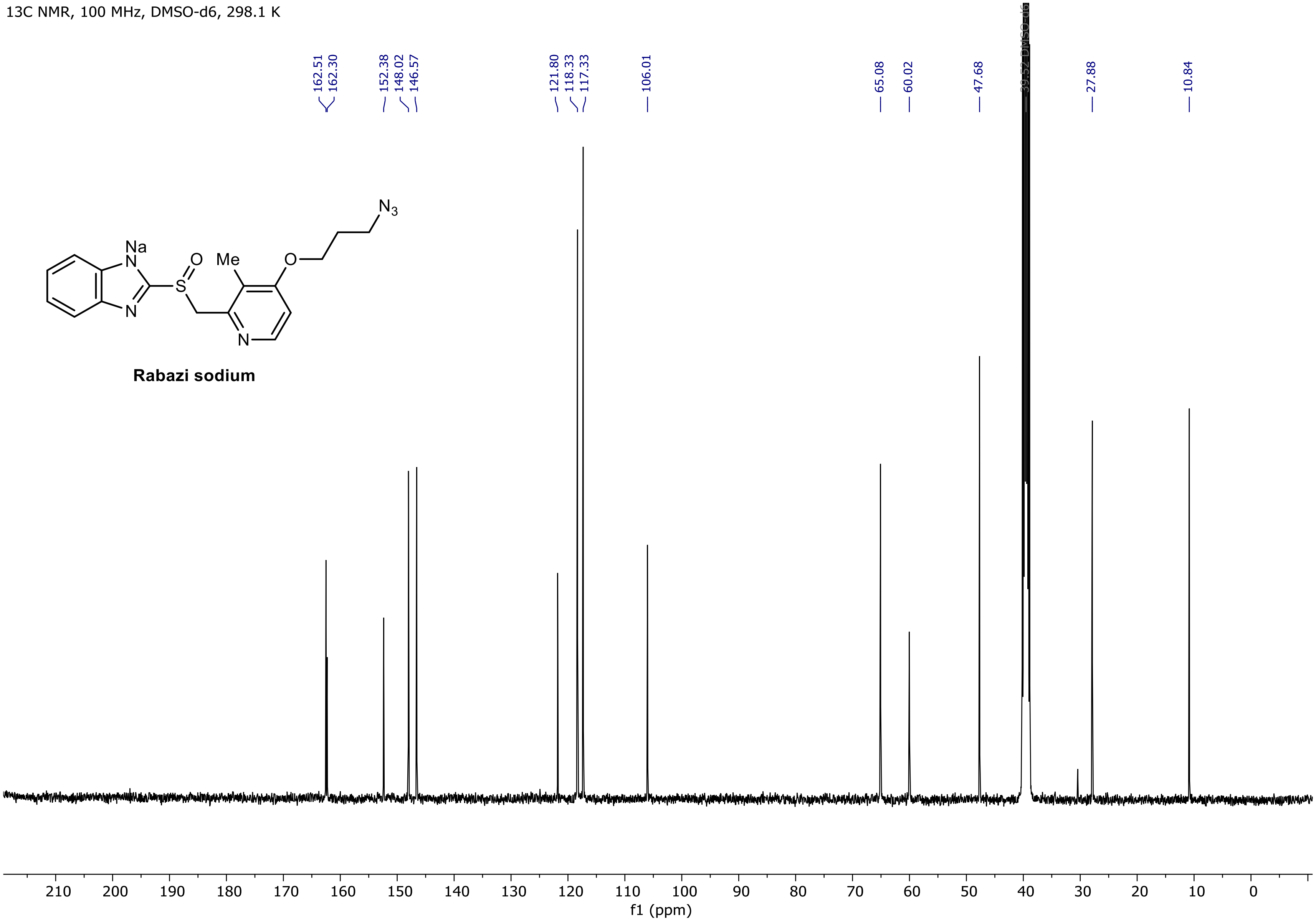
13C NMR, 100 MHz, DMSO-d6, 298.1 K
162.51
162.30
152.38
148.02
146.57
121.80
118.33
117.33
106.01
65.08
60.02
47.68
39.52 DMSO-d6
27.88
10.84
Rabazi sodium
f1 (ppm)

## Supplementary Methods

### Cell culture

HEK293 MSR cells (GripTite™; ThermoFisher) were cultured in high glucose Dulbecco's Modified Eagle Medium (DMEM; Sigma-Aldrich) supplemented with 10% (v/v) Fetal Bovine Serum (FBS; Capricon Scientific), 0.1 mM Minimal Essential Medium non-essential amino acids (Sigma-Aldrich), 100 mg/mL streptomycin (Sigma-Aldrich), 100 U/mL penicillin (Sigma-Aldrich) and 600 µg/mL geneticin (Roth). PACO17 cells[2] were cultured in Primaria tissue culture flasks and dishes using serum-free CSC medium (advanced DMEM/F-12, containing L-glutamine, N-2 supplement, BSA, EGF, bFGF, IGF-I, β-mercaptoethanol, glutathione, heparin, trace elements and lipid mix).

### Plasmid construction for mammalian DENR expression

Expression vectors were generated using the Gibson Assembly Cloning Kit (New England BioLabs). The NEBuilder Assembly Tool was used for primer design. A DNA fragment encoding SBP-DENR* was custom synthesized (GeneArt Strings Synthesis Service, ThermoFisher) and inserted into pcDNA3.1(-).

### Plasmid construction for bacterial DENR expression

Plasmids for bacterial co-expression of MCTS1 and full-length His-DENR were described previously[3]. These include pETDuet-His-DENR-MCTS1, pETDuet-His-DENR(C34A)-MCTS1, pETDuet-His-DENR(C53A)-MCTS1, pETDuet-His-DENR(C37Y)-MCTS1, pETDuet-His-DENR(C34A/C37Y)-MCTS1, pETDuet-His-DENR(C44A/C53A)-MCTS1 and pETDuet-His-DENR(C34A/C37Y/C44A/C53A)-MCTS1. All other vectors were generated using the Gibson Assembly Cloning Kit (New England BioLabs) and the NEBuilder Assembly Tool for primer design. DNA fragments encoding His-TEV-DENR* and $DENR^{26\text{-}98}$ were custom synthesized (GeneArt Strings Synthesis Service, ThermoFisher). For bacterial co-expression of MCTS1 and His-TEV-DENR*, His-DENR was excised from pETDuet His-DENR MCTS1 (using EcoRI/BamHI) and replaced with His-TEV-DENR*, to create pETDuet His-TEV-DENR* MCTS1. For bacterial co-expression of MCTS1 and truncated DENR ($DENR^{26\text{-}98}$), as previously used for crystallization[4], the $DENR^{26\text{-}98}$ fragment was first inserted into pET-His1a (kindly provided by Günter Stier, Heidelberg Biochemistry Center), generating pET-His1a-$DENR^{26\text{-}98}$. MCTS1 and the T7 promoter were then amplified from the pETDuet-His-DENR-MCTS1 plasmid and inserted into the pET-His1a-$DENR^{26\text{-}98}$ plasmid, to generate pET His1a-$DENR^{26\text{-}98}$ MCTS1. All plasmids were verified by sequencing. Mutations were introduced using the QuikChange Site-Directed Mutagenesis Kit (Agilent). Mutagenesis primers (Sigma Aldrich) were designed with the QuikChange Primer Design tool (Agilent).

C53A f ATTCTTCTCTAACCATTGTCTAGCTTTAGCAACATCAGGCATATATTCACAG
C53A r CTGTGAATATATGCCTGATGTTGCTAAAGCTAGACAATGGTTAGAGAAGAAT
C44A f AGCAACATCAGGCATATATTCAGCGTACTCTGTTGGTAATGAACAG
C44A r CTGTTCATTACCAACAGAGTACGCTGAATATATGCCTGATGTTGCT
C37A f CAGTACTCTGTTGGTAATGAAGCGACTCCACAATAAAGGACTCG
C37A r CGAGTCCTTTATTGTGGAGTCGCTTCATTACCAACAGAGTACTG
C34A f TAATGAACAGACTCCAGCATAAAGGACTCGAAGTGGGTAATCG

C34A r CGATTACCCACTTCGAGTCCTTTATGCTGGAGTCTGTTCATTA
C44H f GTACTCTGTTGGTAATGAACAGACTCCGGCATAAAGGAC
C44H r GTCCTTTATGCCGGAGTCTGTTCATTACCAACAGAGTAC
C53H f ATTCTTCTCTAACCATTGTCTATGTTTAGCAACATCAGGCATATATTCACAG
C53H r CTGTGAATATATGCCTGATGTTGCTAAACATAGACAATGGTTAGAGAAGAAT

**Purification of recombinant DENR-MCTS1**

For *in vitro* labeling experiments and mass spectrometry, His-DENR (MW 25.2 kDa) and MCTS1 were co-expressed in *Escherichia coli* BL21(DE3) Rosetta™ 2(DE3)pLysS (Novagen). Lysogeny broth (LB)-medium was inoculated with a single colony, incubated overnight at 37°C and then diluted 1:100 in LB (*in vitro* labeling) or TB (MS). Cultures were grown to an OD of 0.6-0.8 at 37°C before induction with 0.4 mM IPTG. Cells were then incubated at 22 °C with vigorous shaking for 20 h before collecting the cells by centrifugation at 4,000 g for 15 min at 4°C. Cell pellets were stored at -80 °C before resuspension in 20 mM HEPES, pH 7.5, 500 mM NaCl, 1 M Urea, 20 mM imidazole. To lyse the cells, FastBreak™ Cell Lysis Reagent (Promega), lysozyme (Roth) and benzonase (Novagen) were added and cells were incubated for 30 min at RT. The cell lysate was cleared through centrifugation. His-tagged proteins were captured on Ni-Sepharose beads (Cytiva) by rotating at 4°C for 30 min. The beads were then washed three times with at least 20 volumes 50 mM Tris, 150 mM NaCl, 15 mM imidazole pH 7.5. Tagged proteins were eluted by incubation with 2 volumes 50 mM Tris, 150 mM NaCl, 300 mM imidazole pH 7.5 for 5 mins. For *in vitro* labeling, elution fractions were reduced with 1 mM DTT at RT for 5 min, then alkylated with 5 mM NEM at RT for 5 min. Elution fractions were desalted two times using Zeba Spin Desalting columns (Thermo Scientific) to remove imidazole and potentially remaining DTT and NEM. The concentration of the DENR-MCTS1 complex was determined by absorption at 280 nm using a NanoDrop spectrophotometer. For DENR mutants, protein concentration was normalized to wild type by quantifying Coomassie-stained SDS-PAGE gels.

For NMR experiments, His-TEV-DENR$^{26\text{-}98}$ (MW: 10.7 kDa) and MCTS1 were co-expressed in Rosetta™ 2(DE3)pLysS (Novagen). LB medium was inoculated with a single colony, incubated overnight at 37°C and then diluted 1:100 in $^{15}$N-labeled M9-medium. Cells were grown, induced, collected and lysed as described above. The cell lysate was cleared through centrifugation and the supernatant was passed through a 0.22 µm syringe filter. His-tagged proteins were captured on Ni-Sepharose beads by rotating at 4°C for 30 min. The beads were then washed one time with 20 volumes pre-wash buffer (20 mM HEPES, 2 M KCl, pH 7.5) and two times with 20 volumes buffer A (20 mM HEPES, 500 mM NaCl, pH 7.5). Tagged proteins were eluted by incubation with 5 volumes of elution buffer (20 mM HEPES, 500 mM NaCl, 300 mM imidazole, pH 7.5) for 5 mins. To remove the affinity-tag, TEV-protease (GenScript) and 1 mM DTT were added to the elution fraction and incubated for 1 h at RT before transfer to a Slide-A-Lyzer 10K MWCO Dialysis cassette (ThermoFisher) and dialysis against 20 mM Tris, 100 mM NaCl, pH 7.5 overnight at 4°C. Dialyzed protein was again incubated with equilibrated Ni-Sepharose beads to remove TEV-protease and the cleaved peptide tag. The flowthrough was concentrated, loaded on a Superdex 200 Increase 10/300 size exclusion column equilibrated with 20 mM Tris, 100 mM NaCl, pH 7.5 and eluted in 0.5 mL fractions using an Äkta pure FPLC system.

## References


1. Adam, W., Bialas, J. & Hadjiarapoglou, L. Kurzmitteilung / Short Communication A Convenient Preparation of Acetone Solutions of Dimethyldioxirane. *Chem. Ber.* **124**, 2377–2377 (1991).
2. Noll, E. M. *et al.* CYP3A5 mediates basal and acquired therapy resistance in different subtypes of pancreatic ductal adenocarcinoma. *Nat. Med.* **22**, 278–287 (2016).
3. Ahmed, Y. L. *et al.* DENR–MCTS1 heterodimerization and tRNA recruitment are required for translation reinitiation. *PLOS Biol.* **16**, 1–21 (2018).
4. Lomakin, I. B., Dmitriev, S. E. & Steitz, T. A. Crystal structure of the DENR-MCT-1 complex revealed zinc-binding site essential for heterodimer formation. *Proc. Natl. Acad. Sci.* **116**, 528–533 (2019).

## Supplementary Figure S1

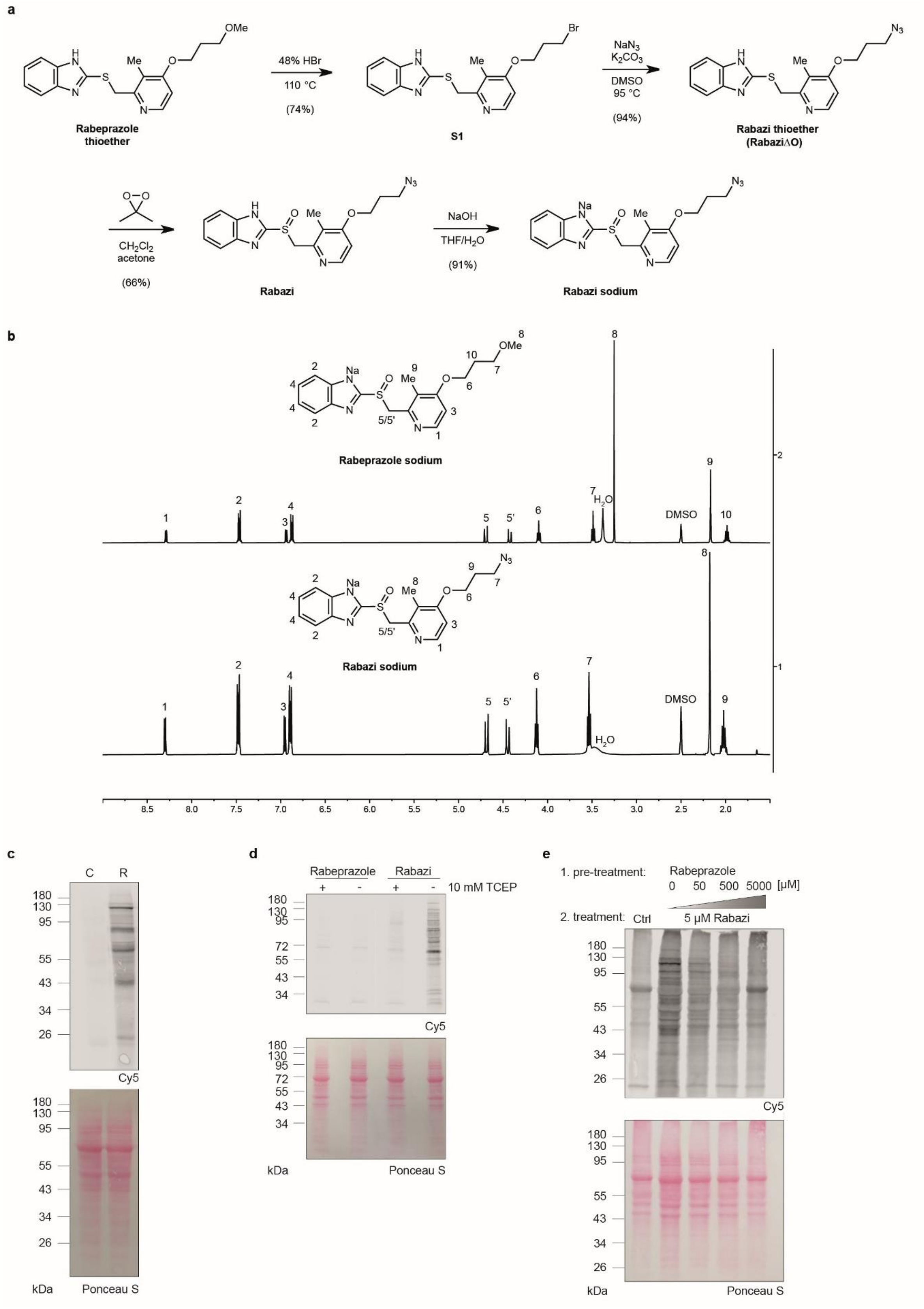

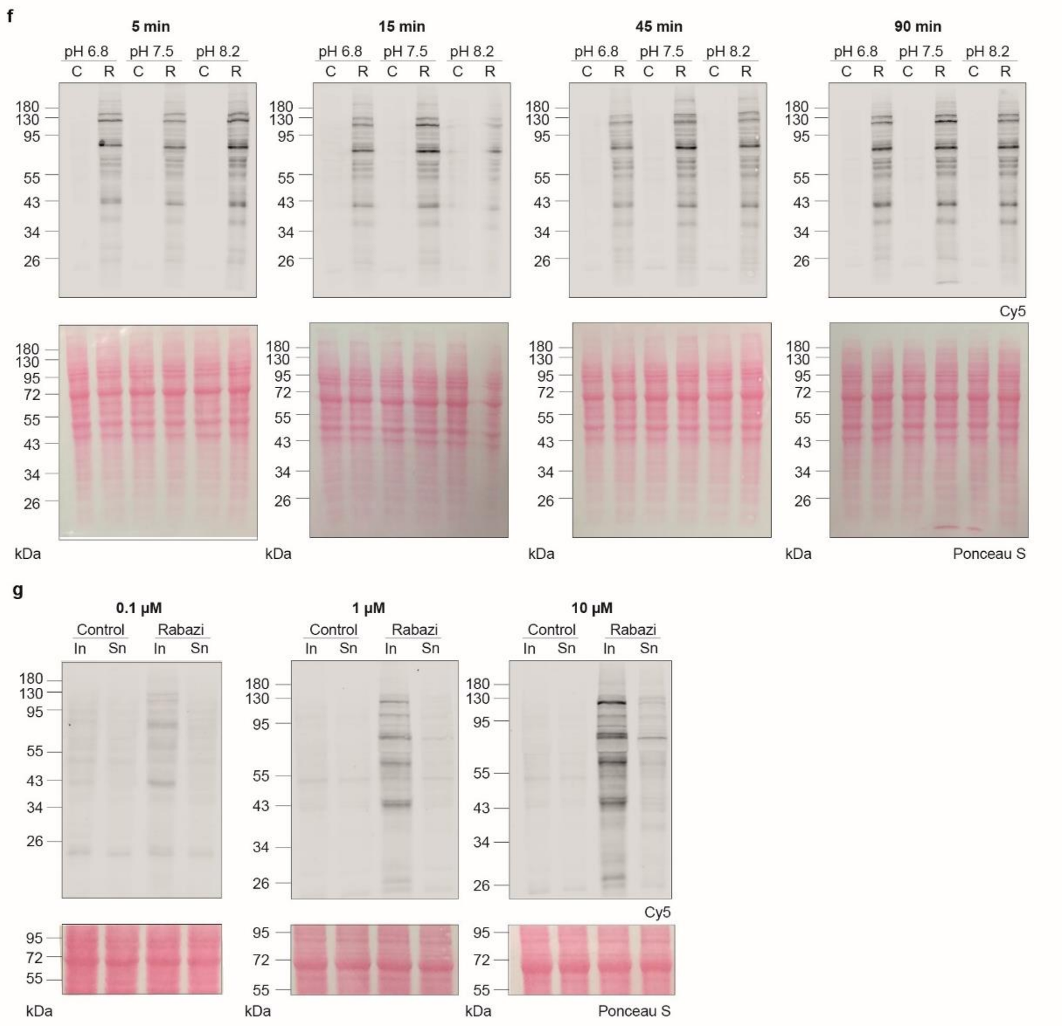


**Supplementary Fig. S1: Synthesis and testing of rabeprazole azide (Rabazi)**

(**a**) Synthesis of rabeprazole azide ('Rabazi').

(**b**) $^{1}$H spectra of rabeprazole (upper panel) and Rabazi (lower panel).

**(c)** Rabazi conjugates to multiple proteins. HEK293 MSR cells were incubated with 10 µM Rabazi (R) or rabeprazole (C).

(**d**) Rabazi-protein conjugates can be reduced by TCEP (10 mM).

(**e**) Rabeprazole and Rabazi compete for conjugating to the same proteins. HEK293 MSR cell lysates were incubated with the indicated concentrations of rabeprazole, followed by incubation with 0 (Ctrl) or 5 µM Rabazi. Free thiols were blocked with NEM and samples concentrated to remove excess reagents. Rabazi-labeled proteins were visualized by SPAAC with DBCO-Cy5.

(**f**) Rabazi conjugation to proteins is independent of medium pH. HEK 293 MSR cells were treated with 50 µM of rabeprazole (C) or Rabazi (R) for the indicated times.

(**g**) Enrichment of Rabazi-conjugated proteins for mass spectrometry analysis. HEK293 MSR cells were treated with the indicated concentrations of rabeprazole or Rabazi for 90 min. Samples before (In) and after (Sn) bead coupling were clicked to DBCO-Cy5.

Blots in c-g are presentative of *n* = 3 independent experiments.

**Supplementary Figure S2**

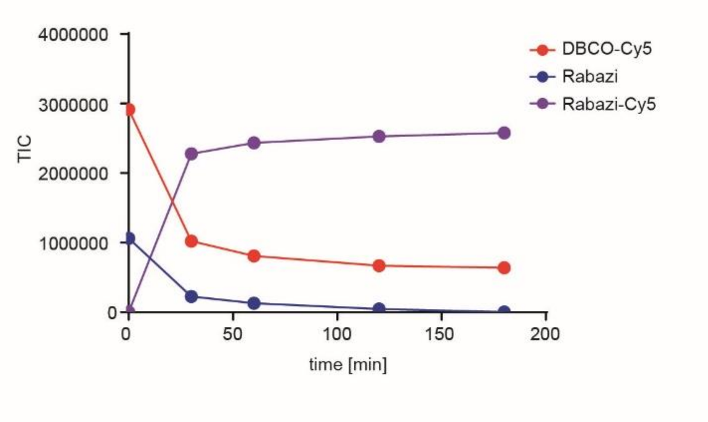


**Supplementary Fig. S2: Monitoring the reaction between Rabazi and DBCO-Cy5**
LC-MS analysis of the click-reaction between DBCO-Cy5 and Rabazi. Total ion count (TIC) of both educts (DBCO-Cy5 and Rabazi) and product (Rabazi-Cy5) over reaction time.

**Supplementary Figure S3**

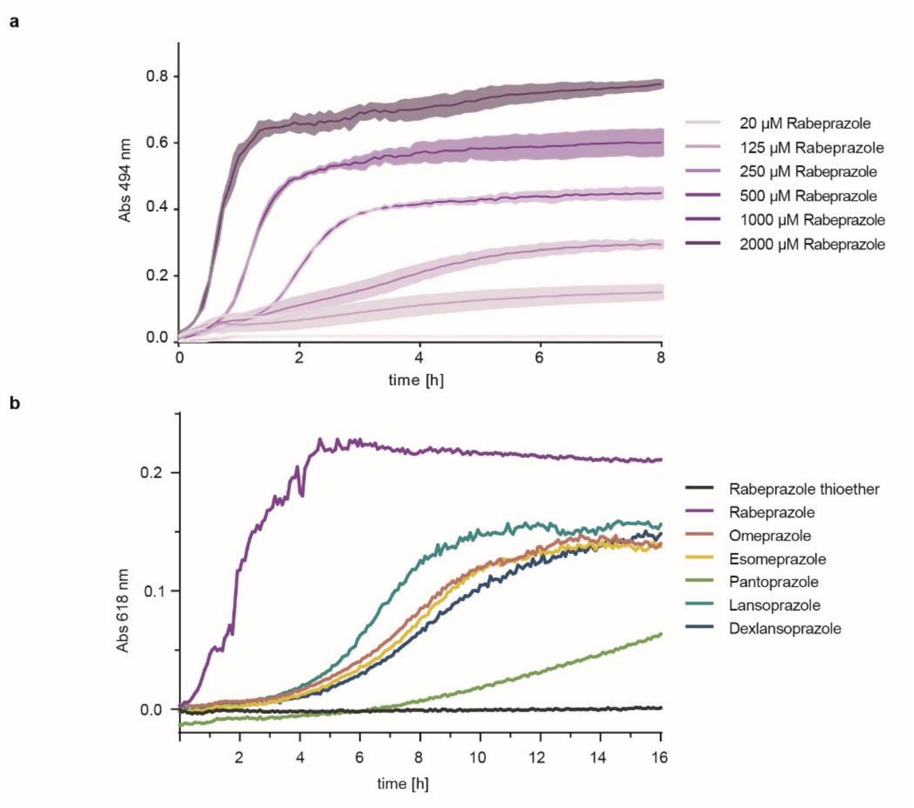


**Supplementary Fig. S3: PPI-induced zinc release from DENR**

(**a**) Release of $Zn^{2+}$ ions from His-DENR* (7.5 µM) in response to different concentrations of rabeprazole, as monitored by chelation with 4-(2-pyridylazo)resorcinol (PAR; 100 µM). Based on the mean of $n$ = 3 (250 µM Rab: $n$ = 2) technical replicates. Shading indicates SD.

(**b**) Release of $Zn^{2+}$ ions from His-DENR* (5.625 µM) in response to all six FDA-approved PPIs, as monitored by chelation with Zincon (100 µM). Rabeprazole thioether serves as a negative control. Based on the background-subtracted mean of $n$ = 3 technical replicates.

**Source data for Supplementary Figure S1C**

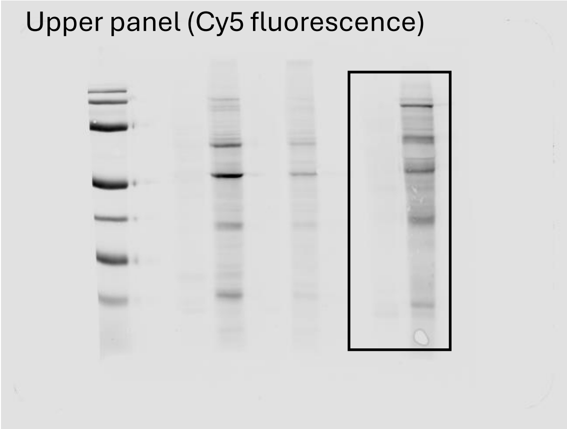


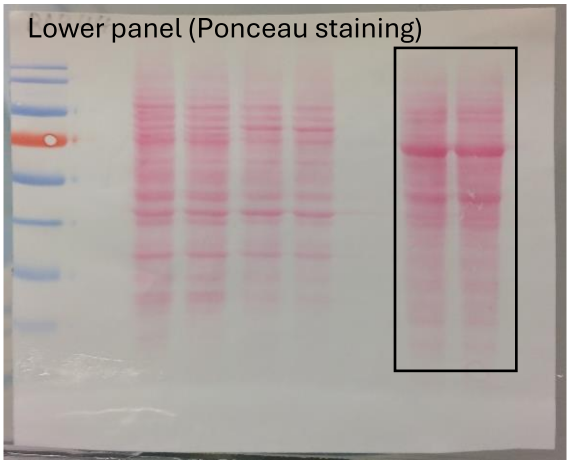

**Source data for Supplementary Figure S1D**

Upper panel (Cy5 fluorescence)

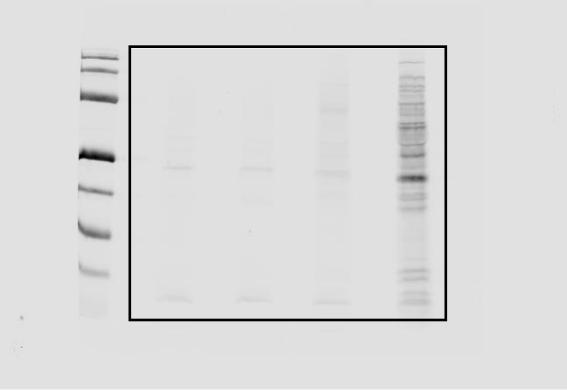

Lower panel (Ponceau staining)

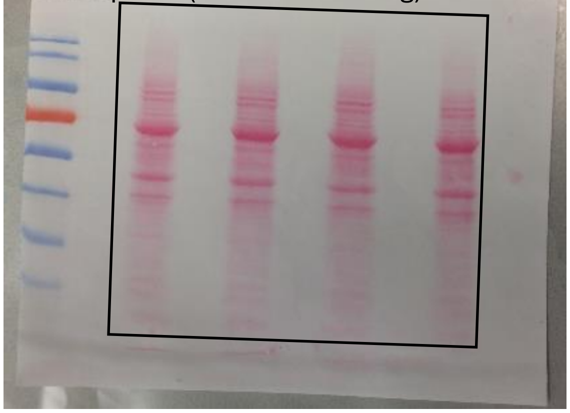

**Source data for Supplementary Figure S1E**

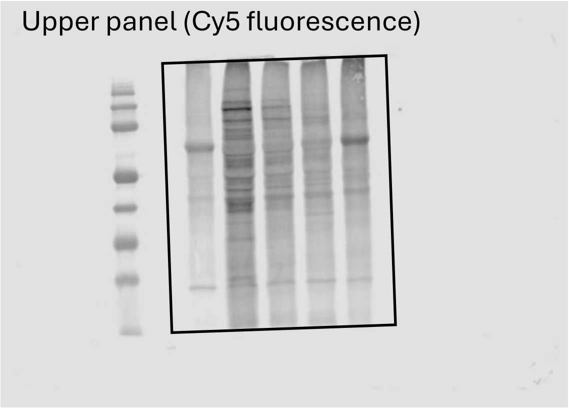


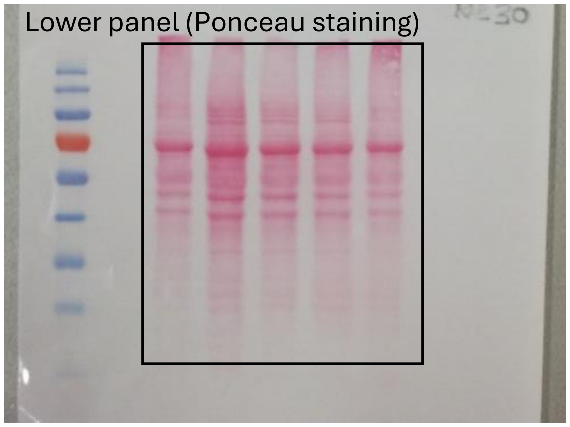

**Source data for Supplementary Figure S1F**

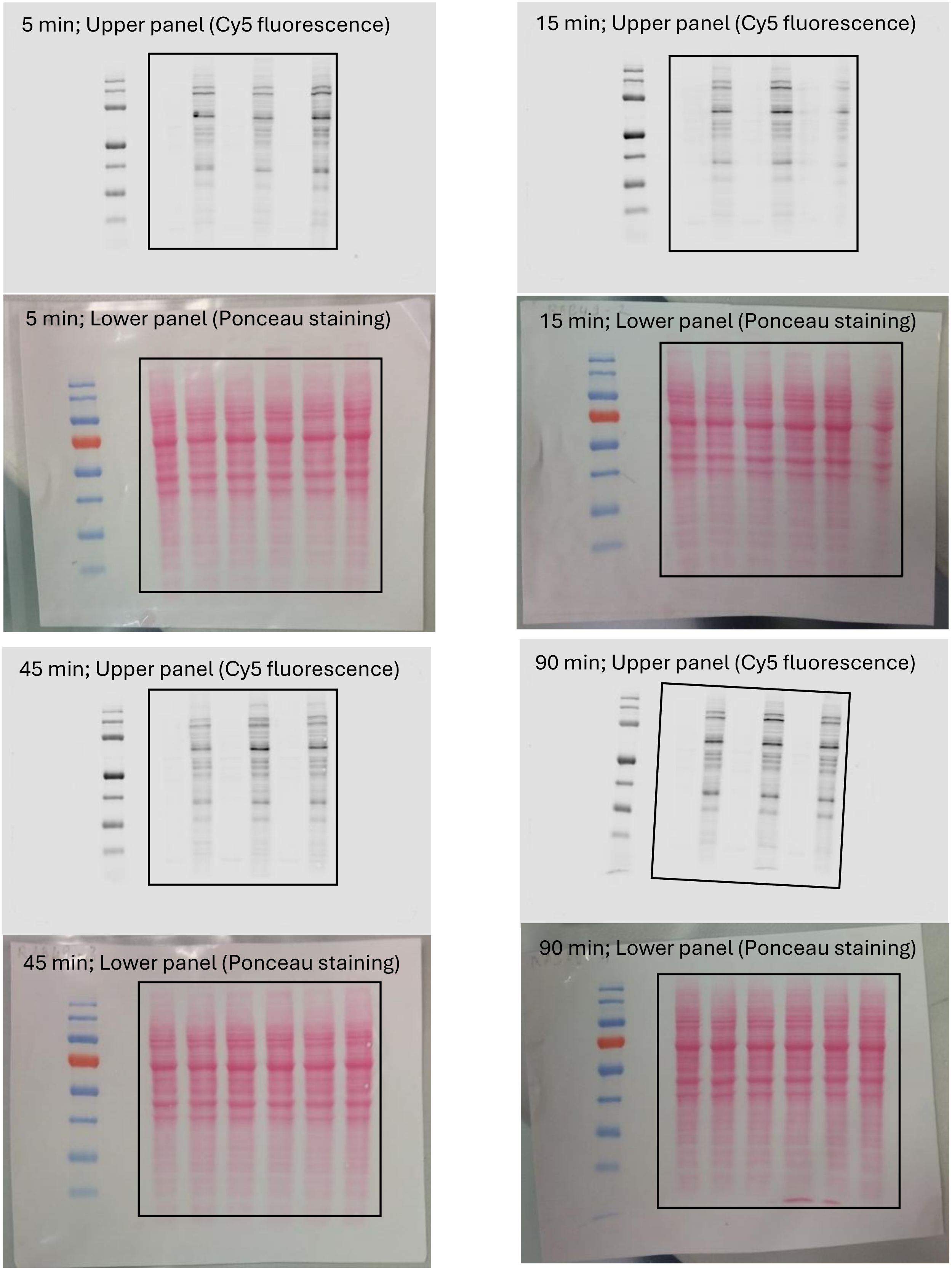

**Source data for Supplementary Figure S1G**

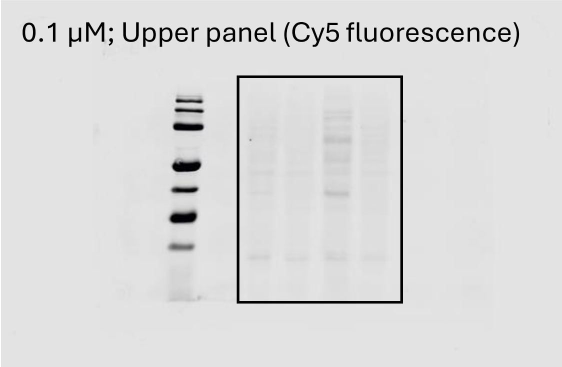


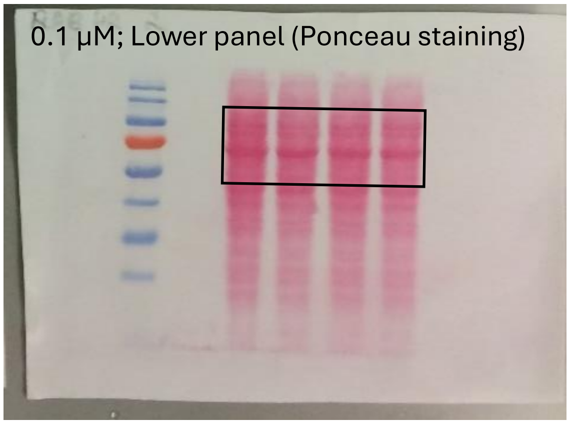


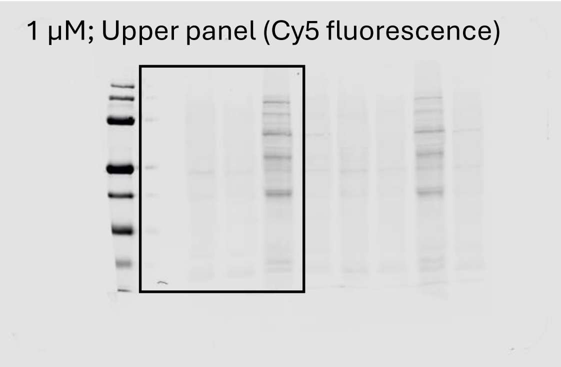


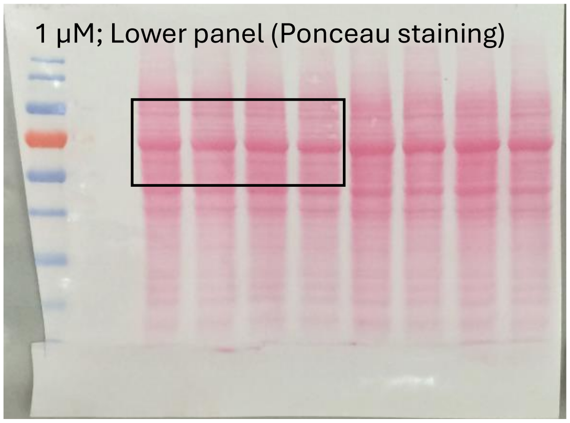


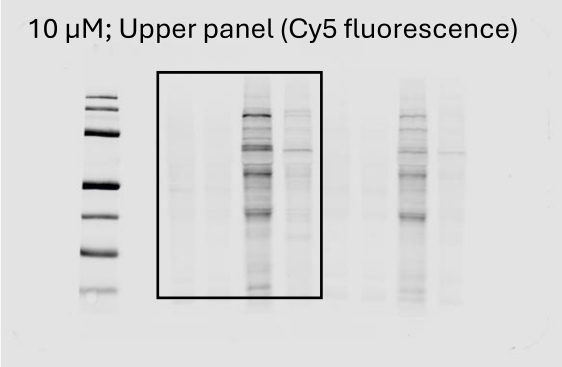


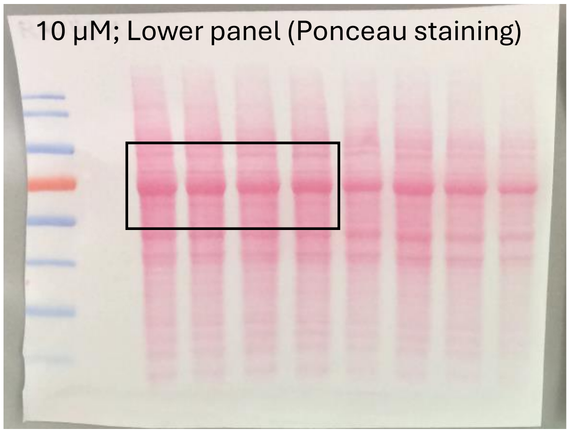

Supplement: Supplementary file 1 — Supplementary Figs. 1–3, Methods and Synthesis notes. [file 41557_2025_1745_MOESM1_ESM.pdf]
